# Highly Viscoelastic Rubber Extrusion: Evolution and Future Perspectives—A Review

**DOI:** 10.3390/polym18161950

**Published:** 2026-08-09

**Authors:** Shixiong Chen, Yancai Sun, Duwei Huang, Jiazhi Yang, Yanbin Ding, Chenbin Lin, Wenzhong Deng

**Affiliations:** 1 School of Mechanical Engineering, Guangxi University, Nanning 530004, China; csxi00@163.com; 2 Guangxi Region Precious Metal Materials Advanced Process Research Center, Guilin University of Aerospace Technology, Guilin 541004, China; 2024025@guat.edu.cn (Y.S.); huangduwei@foxmail.com (D.H.); jiazhi.yang@guat.edu.cn (J.Y.); 3 University Engineering Research Center of Non-Standard Intelligent Equipment and Process Control Technology, Guilin University of Aerospace Technology, Guilin 541004, China; 4 Guangxi Key Laboratory of Special Engineering Equipment and Control, Guilin University of Aerospace Technology, Guilin 541004, China; 5 School of Mechanical and Electrical Engineering, Guilin University of Electronic Technology, Guilin 541004, China; myb905@126.com; 6 School of Mechanical, Naval Architecture and Ocean Engineering, Beibu Gulf University, Qinzhou 535011, China; lcb_0312@163.com

**Keywords:** rubber extrusion, highly filled rubber, nonlinear rheology, constitutive modeling, multiphysics modeling, digital twins

## Abstract

Rubber extrusion has evolved through overlapping advances in equip design, rheological characterization, numerical modeling, sensing, and control. This structured critical narrative review synthesizes 180 sources published from 1972 to 2026, assembled through iterative keyword searching and backward and forward citation tracing. The conventional three-zone theory of solid conveying, compression, and metering is used as a bounded analytical framework, while the literature is organized into four overlapping analytical periods spanning empirical design, constitutive and numerical modeling, engineering-scale simulation, and multiphysics and data-enabled methods. Evidence is distinguished among direct rubber-extrusion validation, rubber-material or rheological studies, transferable general polymer extrusion studies, and enabling computational, sensing, or control research. Five persistent challenges are identified: formulation-dependent nonlinear rheology; incomplete representation of filler-network evolution and wall slip; limited cross-machine and cross-formulation validation; high computational cost; and the lack of standardized datasets and reporting protocols. Digital twins, physics-informed neural networks, and neural operators are promising but remain insufficiently validated for industrial rubber extrusion. Priorities include transparent benchmark datasets, evidence-graded, uncertainty- and latency-aware validation, multimodal sensing, transferable reduced-order and learned models, and measurable sustainability indicators. Together, these priorities define a validation-oriented roadmap for more reliable, transferable, and sustainable rubber-extrusion modeling and control.

## 1. Introduction

Rubber extrusion is one of the core processes in modern rubber product manufacturing and is widely used in the production of critical components such as tires, hoses, sealing strips, and sealing profiles [1]. Since the development of early extruders in the nineteenth century, equipment design, rheological analysis, numerical modeling, sensing, and control have advanced in overlapping rather than strictly sequential ways. Empirical practice, analytical models, computational fluid dynamics (CFD), and data-driven tools continue to coexist, so the discussion below identifies changes in dominant research emphasis without treating digital methods as the inevitable replacement for established engineering practice [2,3].

As a typical non-Newtonian viscoelastic fluid and highly filled composite material, rubber exhibits processing rheological behavior that differs significantly from that of general-purpose thermoplastic polymers [4]. The incorporation of fillers with high specific surface areas, such as carbon black and silica, imparts highly complex nonlinear rheological characteristics to the compound [5,6]. These characteristics, combined with the elevated temperatures, high shear rates, and pressures inherent to the extrusion process, considerably amplify process control uncertainty [7]. The dynamic topological evolution of the filler–matrix network, the temperature- and shear-dependent viscosity behavior, and complex thermomechanical coupling effects collectively render traditional single-variable optimization strategies inadequate for the precision manufacturing of high-performance products [8]. This inherent complexity has been the fundamental driving force behind the continuous evolution of rubber-extrusion technology over the past century.

Existing reviews provide important but distinct coverage. Reference [1] presents a systematic and bibliometric account of polymer extrusion in tire manufacturing; Reference [2] surveys optimization in polymer processing; Reference [4] focuses on rubber rheology in tire-tread extrusion; Reference [9] reviews extrusion modeling across polymer systems; Reference [10] concentrates on computer-aided design and optimization of profile dies; and Reference [11] addresses unified modeling of twin-screw extrusion. These studies establish the broader context but do not provide an evidence-graded synthesis centered on highly filled, highly viscoelastic rubber across feed transport, filler-sensitive rheology, equipment architecture, coupled simulation, sensing, control, and sustainability. Table 1 therefore positions the present review against the most closely related surveys rather than asserting the research gap without comparison.

Accordingly, the contribution of this review is interpretive rather than taxonomic. First, it separates rubber-specific evidence from results transferred from general polymer extrusion and generic computational methods. Second, it evaluates where the classical three-zone theory is useful and where equipment-specific descriptions are required. Third, it organizes technological developments into four overlapping analytical periods anchored to identifiable scientific and industrial indicators. Fourth, it connects material rheology, equipment, modeling, sensing, control, and sustainability through a validation-oriented and measurable research roadmap.

To implement these contributions, this review uses two complementary organizing devices. Four overlapping analytical periods represent shifts in dominant research emphasis, whereas a bounded three-zone theory organizes selected physical questions in conventional single-screw and cold-feed extrusion. Section 2 describes the corpus construction, evidence classification, periodization criteria, and methodological limitations. Section 3 then defines the scope and applicability limits of the three-zone theory, before Section 4, Section 5, Section 6 and Section 7 examine the four analytical periods.

## 2. Review Methodology

This study was conducted as a structured critical narrative review. Its objective was to synthesize the development of rubber-extrusion technology while distinguishing direct rubber-extrusion evidence from rubber-material studies, transferable polymer-extrusion research, and enabling methodological studies.

### 2.1. Information Sources and Search Strategy

The retained bibliographic corpus comprises the 180 sources cited in this review and covers publications from 1972 to 2026. Historical milestones before 1972 were reconstructed from later scholarly reviews and monographs and are used only to provide contextual chronology. The publication counts, evidence classes, and corpus-level analyses reported in this review therefore refer specifically to the retained literature published between 1972 and 2026. The literature was assembled iteratively through keyword-based searches, backward reference checking, and forward citation tracing from key studies and closely related reviews. Web of Science Core Collection and Scopus were used as the principal bibliographic databases, while ScienceDirect and Google Scholar were used for supplementary retrieval and citation tracing. The final reference list was checked and updated on 31 July 2026.

Search concepts were organized into three groups: material terms, including rubber, elastomer, natural rubber, ethylene propylene diene monomer (EPDM) rubber, styrene-butadiene rubber (SBR), nitrile butadiene rubber (NBR), uncured rubber, and highly filled rubber; process terms, including extrusion, extruder, screw conveying, single-screw, twin-screw, die flow, die swell, and co-extrusion; and method terms, including rheology, constitutive modeling, viscoelasticity, wall slip, computational fluid dynamics (CFD), numerical simulation, multiphysics, digital twin, machine learning, sensing, and process control. Material and process terms formed the principal search structure, while method terms were added for topic-specific searches.

Sources were retained when they provided direct evidence concerning rubber or elastomer extrusion, relevant material or rheological characterization, applicable modeling or numerical methods, or enabling sensing and control approaches. Broader thermoplastic and general polymer studies were included only when a clear and explicitly stated transfer pathway to highly filled elastomer processing could be identified. Studies with only generic polymer relevance and no identifiable connection to rubber-extrusion mechanisms, modeling requirements, or process operation were not retained.

The reporting structure was informed by selected transparency principles of the Preferred Reporting Items for Systematic Reviews and Meta-Analyses (PRISMA) 2020 statement [12]. However, because the review was assembled iteratively and a complete record-level search history, deduplication log, and prospective protocol were not retained, it is not presented as a PRISMA-compliant systematic review, and a numerical PRISMA flow diagram is therefore not provided.

### 2.2. Eligibility and Screening Criteria

Eligible sources included peer-reviewed journal articles, review articles, and authoritative scholarly monographs that contributed to rubber or elastomer extrusion, extrusion-relevant rheology, screw conveying and mixing, die flow, constitutive or multiphysics modeling, process sensing and control, or enabling methods with explicit relevance to rubber extrusion.

Sources were excluded when they addressed vulcanization, molding, or compounding without extrusion-relevant analysis; reported only formulation or final-product properties; were conference abstracts, patents, theses, non-peer-reviewed notes, or promotional materials; duplicated a more complete journal publication; or could not be related explicitly to the scope of the review.

**Figure 1:**
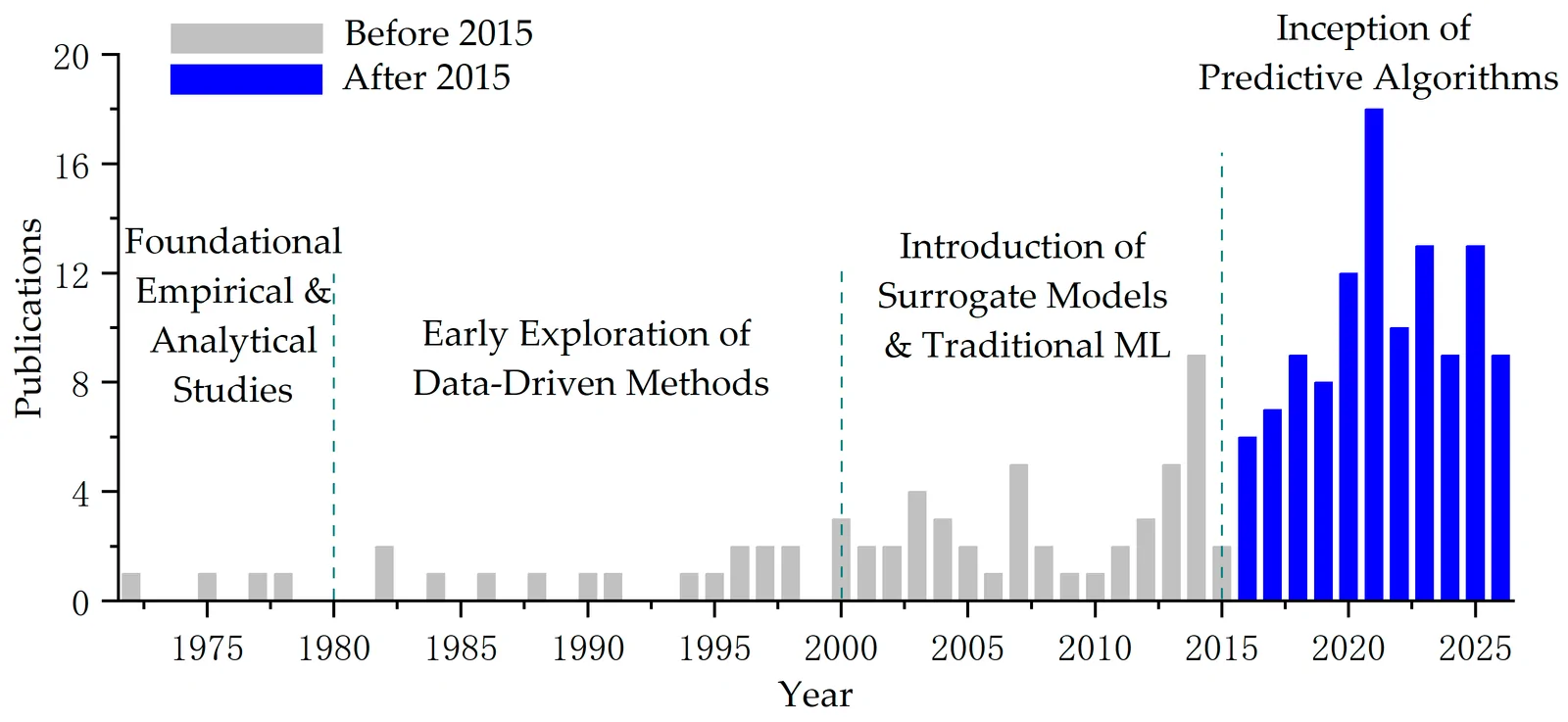
Annual publication distribution of rubber-extrusion references in this study (1972–2026). Original figure prepared by the authors from the final bibliographic corpus. The counts describe the literature analyzed in this review rather than an exhaustive bibliometric representation of the entire field.

Titles and abstracts were first assessed for topical relevance. Full texts were examined when necessary to determine the material system, equipment type, modeling contribution, validation basis, and transferability. Where multiple publications reported substantially overlapping work, the most complete peer-reviewed account was prioritized. The final corpus contains 180 cited sources but is not claimed to be exhaustive. The annual publication distribution of the final corpus is summarized in Figure 1.

### 2.3. Evidence Classification and Data Synthesis

The retained literature was interpreted using four evidence classes: E1, direct rubber-extrusion evidence supported by experimental, pilot-scale, or industrial validation; E2, rubber-material or rheological evidence without full-extruder validation; E3, general polymer or thermoplastic extrusion evidence with an explicit transfer rationale; and E4, enabling computational, sensing, machine-learning, or control research without direct rubber-extrusion validation. These classes indicate the proximity of the evidence to rubber-extrusion application rather than the overall methodological quality of individual studies.

The synthesis considered, where reported, the material formulation, extruder type and scale, operating conditions, constitutive assumptions, wall-slip and filler-network treatment, thermal or reaction coupling, calibration and validation methods, reported performance metrics, computational cost, and evidence class. Numerical comparisons were treated as qualitative or order-of-magnitude indications unless the studies used sufficiently comparable materials, geometries, boundary conditions, and validation metrics.

The retained literature was additionally organized into four overlapping analytical periods. Period I, predominant before the 1980s, represents empirical design, equipment development, and early analytical descriptions. Period II, increasing from the 1980s to the 2000s, represents the broader use of rheological constitutive equations, reduced-dimensional analyses, and early CFD. Period III, increasing from the 2000s to 2015, represents greater use of rubber-specific characterization, engineering-scale CFD, optimization, and multiscale concepts. Period IV, increasing after 2015, represents greater attention to multiphysics coupling, online sensing, digital twins, and data-enabled methods. These labels identify shifts in dominant research emphasis rather than discrete chronological boundaries or technological replacement. The working boundaries were anchored using changes in constitutive modeling, computational capability, and sensing and control maturity, as summarized in Table 2. Direct industrial evidence remains uneven in Period IV, particularly for physics-informed and operator-learning methods.

**Figure 2:**
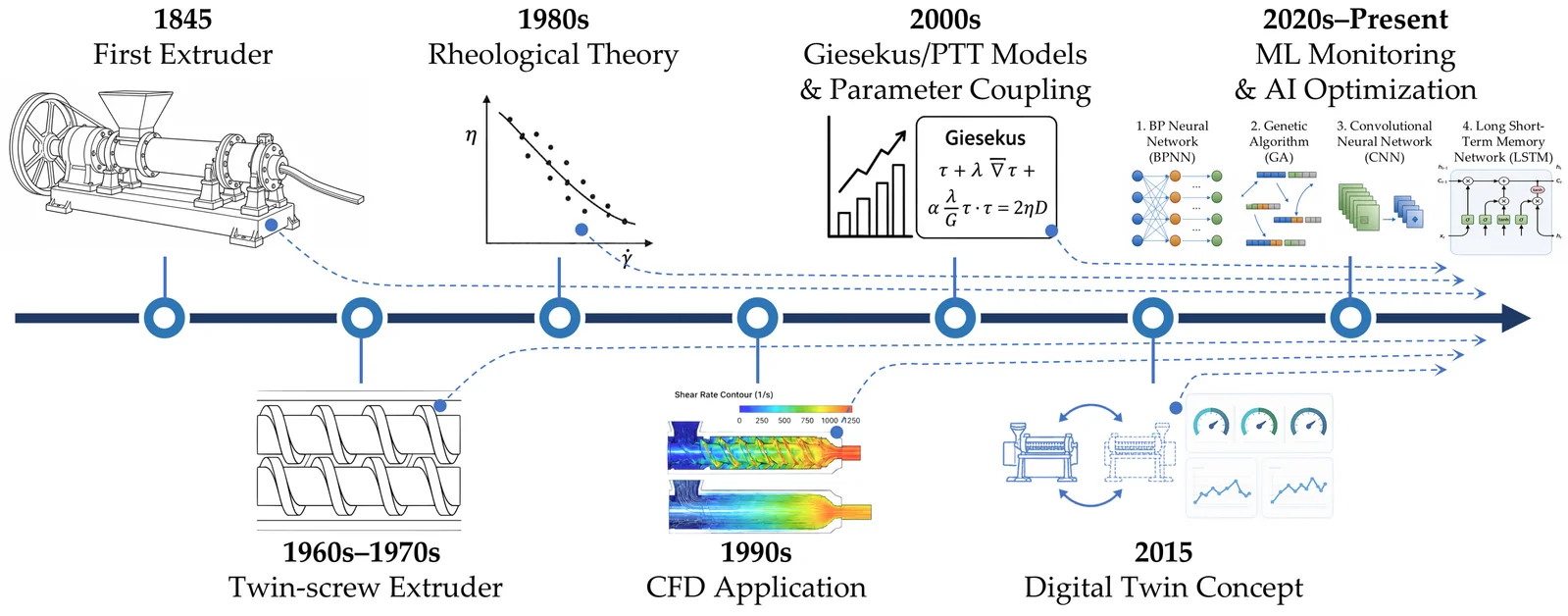
Timeline of the evolution of rubber-extrusion technology. Period boundaries indicate shifts in the dominant mode of inquiry; the approaches shown continue to coexist in current practice, and the transitions between them are gradual rather than abrupt. Original schematic prepared by the authors. Abbreviations: PTT, Phan–Thien–Tanner; ML, machine learning; AI, artificial intelligence.

Two complementary organizing devices were then used in the synthesis. The overlapping periods describe changes in research emphasis, whereas the bounded three-zone theory organizes selected physical questions in conventional single-screw and cold-feed equipment. Neither device implies a universal sequence, and twin-screw elements, dies, sensing, digital twins, and control are treated using equipment- or system-specific descriptions. Figure 2 provides the analytical map used to organize the subsequent historical synthesis and should be read as a map of coexistence rather than as a quantitatively derived or universal sequential-stage model.

Considerable heterogeneity in formulation, equipment, scale, modeling assumptions, instrumentation, and reported metrics prevents meaningful pooled estimates and universal model rankings. In addition, the absence of a complete record-level search history means that the corpus should not be interpreted as exhaustive. Language and publication bias, limited access to industrial data, incomplete reporting in older studies, and reliance on transferred evidence further constrain the generalizability of the conclusions.

## 3. Scope and Theoretical Foundations of the Three-Zone Theory in Rubber Extrusion

**Figure 3:**
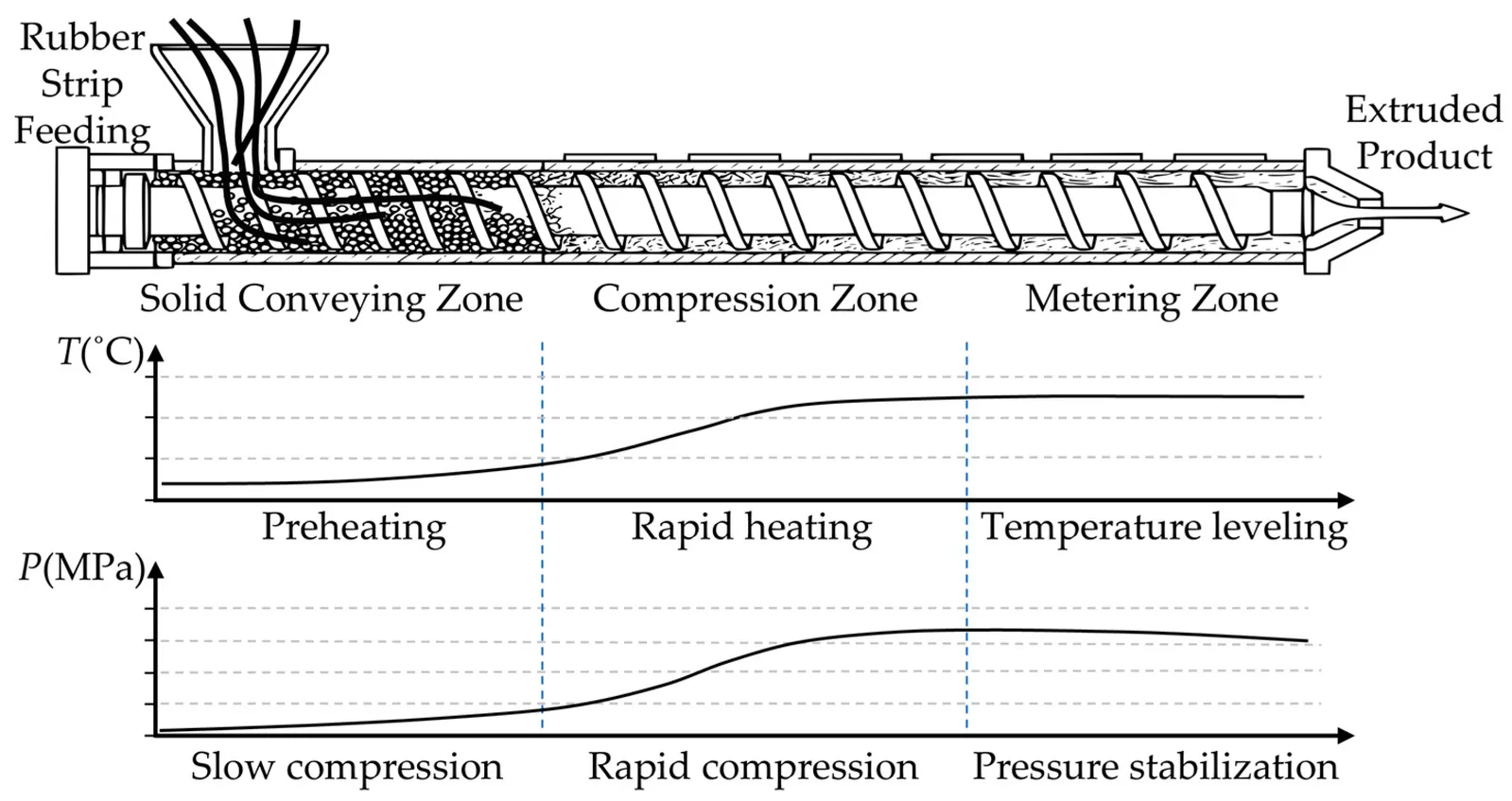
Schematic representation of the three-zone theory and the corresponding temperature and pressure profiles in rubber extrusion. Original schematic prepared by the authors. Axes are qualitative and not to scale; the pressure and temperature profiles are indicative and are not derived from measurement.

Based on differences in material state and dominant mechanisms, a conventional plasticating single-screw extruder is often described in terms of solid conveying, compression or plasticization, and metering (Figure 3). For rubber, these labels are functional analogies rather than fixed period boundaries: cold-fed strips deform and adhere, the material does not undergo crystalline melting, and wall slip, filler-network evolution, and scorch can affect more than one region. The description is therefore used as a diagnostic framework for feeding stability, thermomechanical history, and homogenization before the die, not as an architecture-independent governing theory [13,28].

### 3.1. Solid Conveying Zone

In the solid conveying zone, rubber compounds are subjected to complex frictional dynamics driven by screw thrust and barrel confinement. Feeding efficiency is determined by the interplay between friction coefficients, screw geometry, and feed rates. The foundational understanding of solids conveying in screw extruders was significantly advanced by modified isothermal models [13]. Based on the classical solid conveying theory, the solid conveying rate ($Q_{s}$) is described by:

(1) $$Q_{s}=\pi^{2}D^{2}NH\left(1-\frac{ne}{\pi D}\right)\cos\theta \frac{\tan\phi \tan\theta }{\tan\phi +\tan\theta }$$

where D is the screw diameter, H is the channel depth, N is the rotational speed, n is the number of flights, e is the flight width, θ is the helix angle, and ϕ is the solid conveying angle. This equation highlights the critical role of ϕ in conveying efficiency, a parameter determined primarily by the relative magnitudes of the barrel friction coefficient ($f_{b}$) and the screw root friction coefficient ($f_{s}$). Research has shown that the friction at the pellet–barrel interface is a dominant factor governing the granular transport and pressure buildup [29]. Unlike rigid thermoplastics, the high adhesiveness of rubber makes $f_{b}$ and $f_{s}$ highly sensitive to local temperature and surface roughness; this is particularly evident in the viscoelastic squeezing mechanics of cold-feed rubber strips [30]. At excessive rotational speeds, especially under conditions of backpressure, the tendency for slippage or inefficient conveying intensifies [31]. Experimental investigations at high screw speeds confirm that surface conditions critically influence these transition behaviors [31]. Conversely, insufficient speed or improper feeding regimes lead to unstable flow and starvation [32]. Consequently, early screw designs prioritized geometric optimizations aimed at maximizing ϕ.

It should be noted that the classical theory faces significant applicability boundaries when applied to cold-feed rubber extrusion. Originally developed for thermoplastic pellets, the theory assumes that the solid plug behaves as a rigid body and that the interfacial shear traction obeys Coulomb’s law; that is, it is proportional to the local normal pressure with a constant friction coefficient. These assumptions do not fully capture the behavior of deformable and adhesive rubber feeds. Studies on single-screw plasticating extrusion have long indicated the necessity of accounting for non-plug flow phenomena in the solid conveying zone [14]. More recently, discrete particle simulations have been employed to elucidate the complex mechanisms of solids compaction and conveying, bridging the gap between theoretical models and practical observations [33]. Furthermore, numerical simulations of solids conveying in grooved feed sections have provided deeper insights into the pressure generation mechanisms [34].

Equation (1) is obtained by multiplying the available screw-channel cross-sectional area by the axial velocity of a compact solid plug, with the latter determined from a frictional force balance in the helical screw channel [13]. Its native derivation concerns pellet-fed granular solids in a conventional single-screw extruder and assumes steady and isothermal conveying, approximately constant bulk density, rigid-plug motion, and Coulomb friction at the barrel and screw surfaces. Under the Coulomb-friction assumption, the interfacial shear traction is proportional to the local normal pressure, whereas the friction coefficient is treated as constant. The number and width of the flights and the helix angle enter Equation (1) as geometric corrections; however, internal plug deformation, adhesive or pressure-dependent friction, sidewall effects, leakage across the flights, and three-dimensional recirculation are not resolved. Equation (1) is therefore retained as a first-order reference for conventional pellet conveying and should not be applied quantitatively to deformable and adhesive cold-feed rubber strips without rubber-specific calibration or a deformable-solid conveying model.

### 3.2. Compression Zone

Unlike the crystalline melting of thermoplastics, rubber “plasticization” is a complex non-equilibrium transformation involving enhanced polymer segment mobility coupled with filler-network restructuring [35,36,37,38]. Driven by screw shear and barrel heat transfer, this process obeys the energy balance equation for convection, conduction, and viscous dissipation [39]:

(2) $$\rho c_{p}\frac{DT}{Dt}=\nabla \cdot \left(k\nabla T\right)+\eta {\dot{\gamma }}^{2}$$

The role of viscous dissipation in such flows has been highlighted in numerical simulations of plastic fluids, where shear work generates substantial internal heat [40]. In high-viscosity rubber systems, the contribution of viscous dissipation ($\eta {\dot{\gamma }}^{2}$) is relatively high, making the compound highly susceptible to localized shear heating. This is further complicated by exothermic thermal responses, where the coupling of shear flow and heat generation can lead to rapid temperature escalation [41]. The addition of reinforcing fillers further complicates this energy distribution. Although these fillers increase the apparent viscosity, their influence on extrusion rheology varies substantially with mixing temperature [42]. Furthermore, wet continuous mixing studies indicate that controlled thermal management can influence energy input and filler distribution [43].

Equation (2) follows from the local continuum energy balance after retaining heat conduction and viscous dissipation. Unlike Equations (1) and (3), it is explicitly non-isothermal and does not require pellet feeding or rigid-plug transport. In the simplified scalar form $\eta {\dot{\gamma }}^{2}$, the dissipation term is consistent with a Newtonian closure when η is constant and with a generalized-Newtonian approximation when $\eta =\eta (\dot{\gamma },T)$ is specified as a shear- and temperature-dependent apparent viscosity. It does not, however, represent the complete stress power or reversible elastic-energy storage of a differential viscoelastic constitutive model. The equation further assumes a single-phase continuum in local thermal equilibrium, with density, heat capacity, and thermal conductivity treated as constant or prescribed material functions. Screw-flight effects are not represented explicitly and enter only through the velocity, shear-rate, and temperature fields obtained from the associated flow solution. Accordingly, Equation (2) provides a baseline non-isothermal energy balance; wall slip, filler-network evolution, spatially varying properties, reaction heat, and scorch kinetics must be coupled and validated separately when they are significant [41,44].

**Figure 4:**
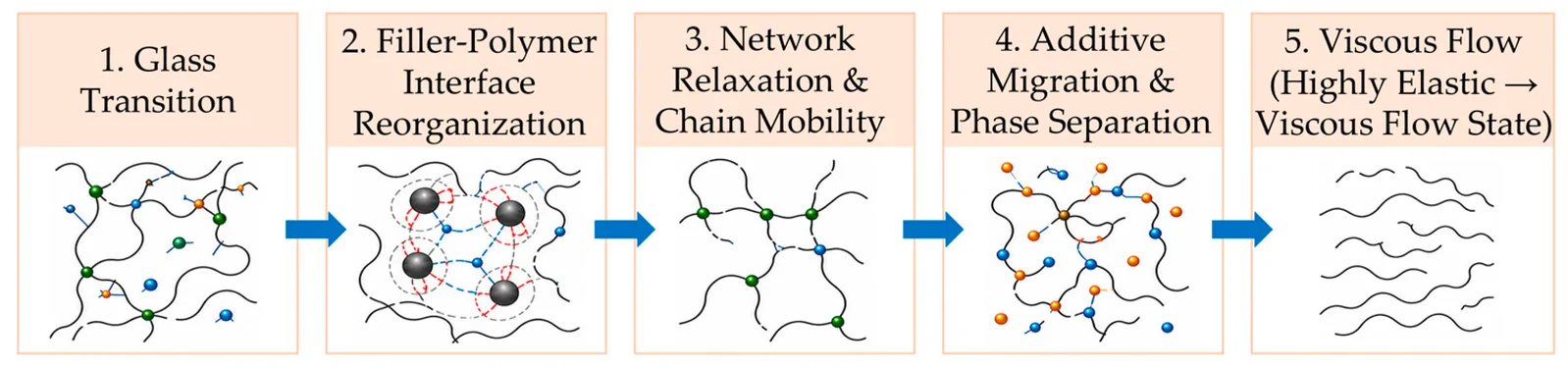
Multi-stage evolution of microstructural and molecular network configurations during the extrusion processing of highly viscoelastic rubber. Original schematic prepared by the authors, summarizing mechanisms reported in [35,36,37,38]. The figure is conceptual: length scales are not to scale and the configurations shown are not derived from imaging data.

A primary multiscale mechanism in this zone is the dynamic disaggregation and re-agglomeration of filler networks (Figure 4). The dispersion state of filler and the polymer–filler interactions are fundamental to the composite’s processing performance [35]. Under high shear fields, filler agglomerates are forced to break down. However, the extent of this breakdown and the resulting rheological behavior are heavily dependent on the filler–filler interaction strength [36]. In regions of lower shear flow, dispersed particles are prone to secondary agglomeration. This confers pronounced thixotropic properties on the compound, which can be characterized by experimental analysis of the viscoelastic properties in filled natural rubber systems [37]. Both linear and nonlinear rheological behaviors of filled rubbers, such as silica-filled systems, reflect this complex structural evolution [38]. This mechanism creates a fundamental trade-off in the processing window: sufficient shear input is required to disperse the filler network, yet the resulting temperature rise must be strictly controlled to prevent thermal oxidation and scorching [35,36,37,38,41].

### 3.3. Metering Zone

The metering zone is tasked with homogenizing the compound’s temperature, viscosity, and filler dispersion via continuous shear mixing, thereby supplying the die with material possessing stable rheological properties. A shallower channel depth is typically used in this zone to intensify shear. Experimental determination of shear-thinning behavior during the extrusion of rubber blends confirms that viscosity decreases significantly with increasing shear rate [45]. The macroscopic volumetric flow rate (Q) results from the superposition of forward drag flow and pressure-driven backflow:

(3) $$Q=\frac{1}{2}\pi^{2}D^{2}NH_{3}\sin\theta \cos\theta -\frac{\pi DH_{3}^{3}{sin}^{2}\theta }{12\eta }\frac{\Delta P}{L}$$

where $H_{3}$ is the channel depth in the metering zone, and ΔP/L represents the axial pressure gradient. To describe this non-Newtonian flow accurately, advanced capillary rheological models expressed by multi-parameter flow equations have been developed to capture the true flow behavior of unvulcanized rubber [46].

Equation (3) is obtained by superposing idealized down-channel drag flow generated by the relative motion of the barrel and screw and pressure-driven backflow in an unwound helical channel. The closed-form expression assumes steady, fully developed, laminar, incompressible, fully filled, and isothermal Newtonian flow with constant viscosity, no wall slip, a shallow channel, and an approximately linear axial pressure gradient. Inertia, channel curvature, cross-channel secondary flow, finite flight-side effects, and flight-tip leakage are neglected. The screw flights are therefore represented only through the idealized channel dimensions and helix geometry rather than through a resolved three-dimensional flow field. For generalized-Newtonian rubber, the drag- and pressure-flow contributions must be recalculated using a shear- and temperature-dependent viscosity, generally through numerical integration. Viscoelastic memory, normal stresses, wall slip, filler-network evolution, strong thermal gradients, and die coupling require a more complete constitutive and CFD formulation. Equation (3) is consequently retained as a limiting historical baseline rather than as a stand-alone design equation for highly filled rubber extrusion [9,11].

Furthermore, the metering zone contributes to the stabilization of temperature, flow, and filler dispersion. Inadequate homogenization or an unsuitable thermal history may promote filler re-agglomeration and surface-quality deterioration, whereas excessive thermomechanical loading may contribute to material degradation; these relationships remain formulation- and process-dependent [35,36,37,38,44,47]. The specific formulation, including curing agents and carbon-black loading, dictates the rheological properties and processing stability of EPDM and similar compounds. Die swell (commonly known as the Barus effect) [48], which occurs as the extrudate exits the die, originates from the relaxation of stored elastic energy and is jointly governed by the die land geometry, shear rate, and the elastic modulus of the compound. The extrudate swell of molten polymers, from fundamental molecular understanding to die design, has been extensively reviewed [49]. Comparative studies have shown that extrudate swell in non-Newtonian fluids differs fundamentally from Newtonian cases, particularly under the influence of gravitational body forces [50]. For rubber compounds specifically, rheological characterization of the die-swell phenomenon is essential for predicting dimensional stability [51]. Detailed investigations into the die-swell behavior of rubber compounds during short-die extrusion [52] and the significant effect of the die angle on the final extrusion swell [53] have further refined the empirical understanding of post-die relaxation. Consequently, the primary objective of the metering zone design is to ensure the compound achieves an optimal microstructural state prior to die entry through precise management of its thermomechanical history.

The metering-zone description therefore provides a useful reference for relating pressure generation, thermal homogenization, rheological stability, and die-entry conditions in conventional single-screw extrusion. Its applicability to cold-feed rubber, twin-screw elements, and system-level sensing or control is qualified systematically in Section 3.4. Section 4, Section 5, Section 6 and Section 7 then examine how equipment design, constitutive modeling, CFD, sensing, and data-enabled methods have progressively addressed these limitations.

### 3.4. Applicability and Limits of the Three-Zone Theory

The native domain of the three-zone theory is a conventional plasticating single-screw extruder with a recognizable axial progression from feeding through thermomechanical conditioning to a relatively homogeneous metering state. In this review it is used as a diagnostic and comparative scaffold, not as an architecture-independent governing theory.

For co-rotating twin-screw machines, conveying can approach positive displacement, kneading blocks generate elongational and back-mixing flows, and the intermeshing region imposes strongly time-dependent deformation histories [54,55,56]. Such systems should be analyzed using element-resolved conveying, kneading, residence-time distribution, and mixing descriptions. Dies, sensing, digital twins, and control are downstream or system-level layers rather than additional screw zones.

For cold-feed rubber, the framework must account for viscoelastic strip deformation, adhesive and pressure-dependent friction, wall slip, shear heating, the absence of crystalline melting, and a scorch-limited thermal history [14,31,33,34,56,57]. These qualifications apply wherever three-zone terminology is used below.

Table 3 makes explicit that die flow, online sensing, digital twins, and intelligent control are coupled downstream or system-level layers rather than additional screw zones. Full-process models should state which mechanisms are solved directly, represented empirically, or omitted, and should replace forced zone mapping with equipment-specific descriptions wherever the analogy breaks down.

## 4. Empirical Design and Equipment Development (Predominant Before the 1980s)

### 4.1. Preliminary Applications and Empirical Design in Tire Production

Early rubber-extrusion development was strongly empirical, particularly in tire and continuous-profile production. Die dimensions were commonly adjusted through trial-and-error to compensate for extrudate swell and post-extrusion shrinkage [15,61,62]. Single-screw extruders dominated the era, but their simple flow-channel geometry provided limited capability for both distributive mixing and dispersive mixing, confining their applicability to relatively simple compounds such as sidewall and tread stocks [72,73].

The matching of formulations and equipment long depended on trial-and-error methods. The industry gradually recognized the strong coupling between rubber compound formulations and screw configurations: for high-viscosity, highly filled systems, geometric modifications such as reducing the screw channel depth were necessary to intensify the shear field. Yet such adaptations proved insufficient. The complex dispersion behavior of carbon black and silica within the rubber matrix, compounded by the nonlinear influence of additives on rheological properties, meant that traditional single-screw extruders consistently fell short of the microscopic homogeneity required for high-performance tire compounds [74,75].

**Figure 5:**
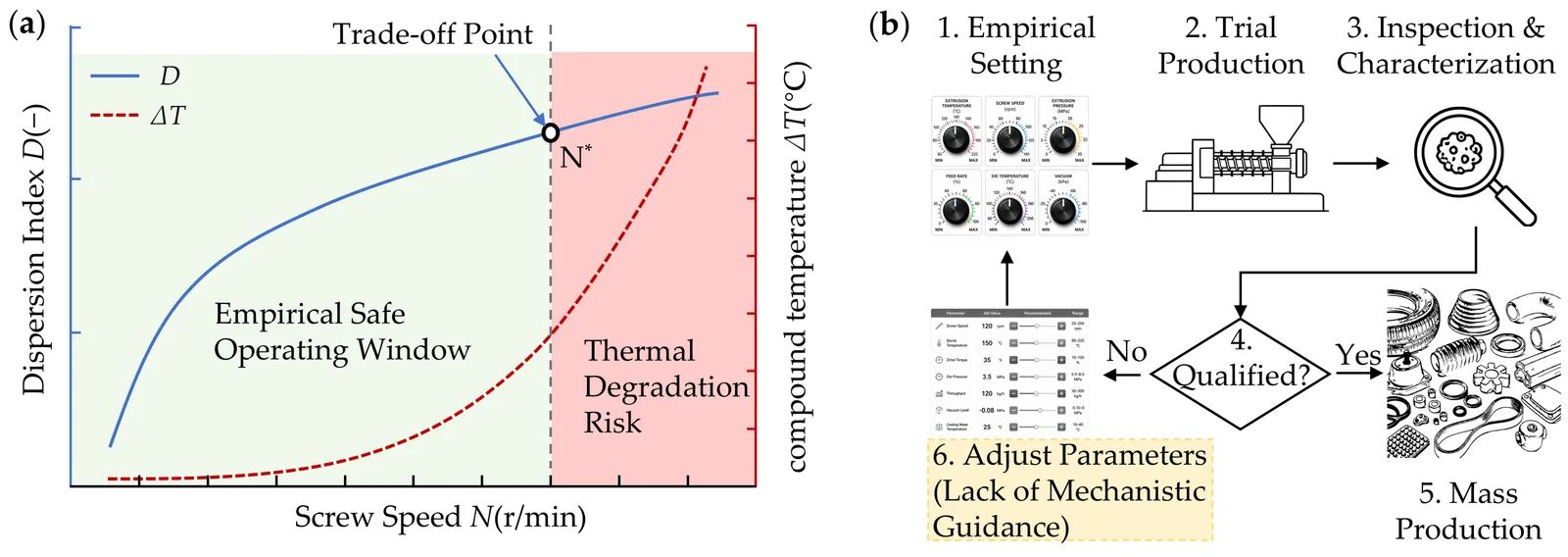
Empirical process–quality relationships and trial-and-error optimization logic during the early development of rubber extrusion: (**a**) schematic diagram of the relationship between screw speed, dispersion index, and compound temperature rise during the initial stage of rubber extrusion, $N^{*}$ denotes the screw speed at the illustrative trade-off point; (**b**) process optimization via early empirical trial-and-error method. Original schematic prepared by the authors. Both panels are qualitative; the axes carry no units, and the curves are illustrative rather than measured.

From a rheological perspective, the characteristic shear-thinning behavior of uncured rubber compounds imposed an implicit constraint on early empirical operations. Increasing screw speed could enhance shear to improve mixing but inevitably exacerbated viscous dissipation, causing excessive temperature rise in the compound and triggering thermal oxidative degradation. Although early engineers empirically identified a “safe window” for process parameters, they lacked a quantitative framework based on rheological constitutive models to elucidate the underlying physical mechanisms (Figure 5a). Even early computational attempts to model single-screw plasticating behavior highlighted the formidable challenge of coupling thermal and fluid dynamics in non-Newtonian systems [72].

### 4.2. The Emergence and Innovation of Twin-Screw Extruders

During the 1960s and 1970s, the industrial adoption of twin-screw extruders expanded the deformation and residence-time histories available in rubber processing. Depending on rotation mode, intermeshing geometry, and element sequence, twin-screw configurations can provide strong distributive and dispersive mixing through repeated shear, elongation, splitting, and recombination [65]. These advantages are configuration- and formulation-dependent and should not be interpreted as evidence that twin-screw equipment universally superseded single-screw rubber extrusion.

Early twin-screw systems nevertheless remained rudimentary in both configuration design and process control. Component geometries were restricted to uniform pitch and simple symmetric or asymmetric arrangements, with no established modular design philosophy. Temperature control typically relied on coarse, segmented barrel heating, while drive systems depended on fixed-frequency alternating-current motors, severely limiting the precision of speed regulation across a wide operating range. Subsequent equipment iterations, such as the development of specialized cold-feed extruders with optimized energy management, would eventually address some of these thermal and control inefficiencies [16]. Of greater significance, the complex non-Newtonian flow and mixing kinetics within the intermeshing zone lacked a rigorous mathematical description. While specialized process description methodologies and screw design software were later developed to bridge this gap [28], early process optimization remained confined to empirical adjustments that varied one parameter at a time—typically rotational speed or barrel temperature.

### 4.3. Process Exploration and Theoretical Gaps

Despite notable progress in equipment iteration and practical processing during this period, the absence of a solid theoretical foundation remained the principal bottleneck constraining further technological advancement.

Engineers possessed a qualitative understanding of how screw speed influenced residence-time distribution (RTD), thermal history, and mixing quality, yet the underlying nonlinear relationships among these variables remained unquantified. Screw speed, feed rate, and screw configuration jointly affect throughput, residence-time distribution, thermal history, and mixing, and these coupled effects influence carbon-black dispersion and compound microstructure [65,74]. Without quantitative theoretical tools, optimization of processes involving multiple coupled parameters was impeded.

Material developments that became increasingly important in subsequent periods further revealed the limitations of early empirical processing. The increasing use of silica, either alone or in hybrid filler systems, has been driven partly by the demand for lower rolling resistance in tire-tread compounds [2,76,77,78]. However, the abundant silanol groups on the silica surface severely degraded its compatibility with the hydrophobic rubber matrix, leading to pronounced filler–filler and filler–rubber network complexities [79]. The addition of silane coupling agents improved interfacial bonding but markedly increased the kinetic complexity of the compounding process [80,81]. The specific interaction mechanisms—ranging from the chemical grafting of coupling agents on the silica surface to the physical reinforcement provided by varying agent functionalities—proved highly intricate [82,83]. These later developments confirmed that the rheological parameters of rubber are not fixed material constants, but dynamic functions governed by formulation, processing history, filler-network morphology, and thermal conditions.

In summary, the period from the early 20th century to the 1980s combined empirical equipment knowledge, early analytical descriptions, and emerging material characterization (Figure 5b). Twin-screw development expanded the available mixing mechanisms, but single- and twin-screw machines continued to serve different processing needs. The limited transferability of operator-led adjustments across compounds and equipment created the demand for rheological models and numerical simulation methods.

## 5. Constitutive Modeling and Early Numerical Simulation (Increasing from the 1980s to the 2000s)

### 5.1. Establishment of the Theoretical Foundations of Rheology

Beginning in the 1980s, advances in polymer rheology and polymer physics strengthened the theoretical basis for rubber extrusion. Empirical models such as the power-law, Carreau, and Herschel-Bulkley equations were introduced into engineering calculations using rheological measurements at different temperatures and shear rates, supporting a gradual shift from trial-and-error toward quantitative analysis. The Giesekus model introduces deformation-dependent tensor mobility and can represent shear-thinning and normal-stress effects under appropriate calibration [17,84]; Zatloukal developed differential viscoelastic equations for steady shear and extensional flow [18,85]. Their usefulness nevertheless depends on parameter identification, flow type, numerical robustness, and validation for the compound and operating range of interest.

Qualitative curve shapes alone do not establish the predictive superiority of a constitutive model. The model families are therefore compared in Table 4 according to the physical phenomena represented, calibration requirements, numerical robustness, computational demand, validation scale, and proximity of the evidence to rubber-extrusion applications.

In the modeling of filled systems, classical suspension-rheology models predict a divergence in viscosity as the filler volume fraction approaches the maximum packing fraction, providing a macroscopic benchmark for interpreting the enhanced non-Newtonian response of highly filled rubbers [5]. However, traditional single-phase rheological models often fail under high shear fields, as they cannot characterize the nonlinear rheological response resulting from the “breakdown–reorganization” dynamics of the filler network. This limitation prompted the introduction of structural parameters into model frameworks to capture the feedback effect of the filler network on energy dissipation [86].

In the late 1990s, the emergence of interfacial chemical rheology further expanded the dimensions of modeling. Research confirmed that surface modification techniques, such as the use of silane coupling agents, not only improved the physical compatibility between silica and the rubber matrix but also significantly altered macroscopic rheological responses—particularly die swell—by weakening the filler-network effect. This prompted a pivotal shift in modeling consensus: high-fidelity extrusion models could no longer be confined to the homogeneous viscosity hypothesis but had to map microscopic parameters, such as the surface modification state of fillers and the evolution of dispersion, to macroscopic rheological predictions [80,81].

### 5.2. Multidimensional Numerical Analysis of Screw Extrusion Flow Fields: From Dimension Reduction to 3D CFD

**Figure 6:**
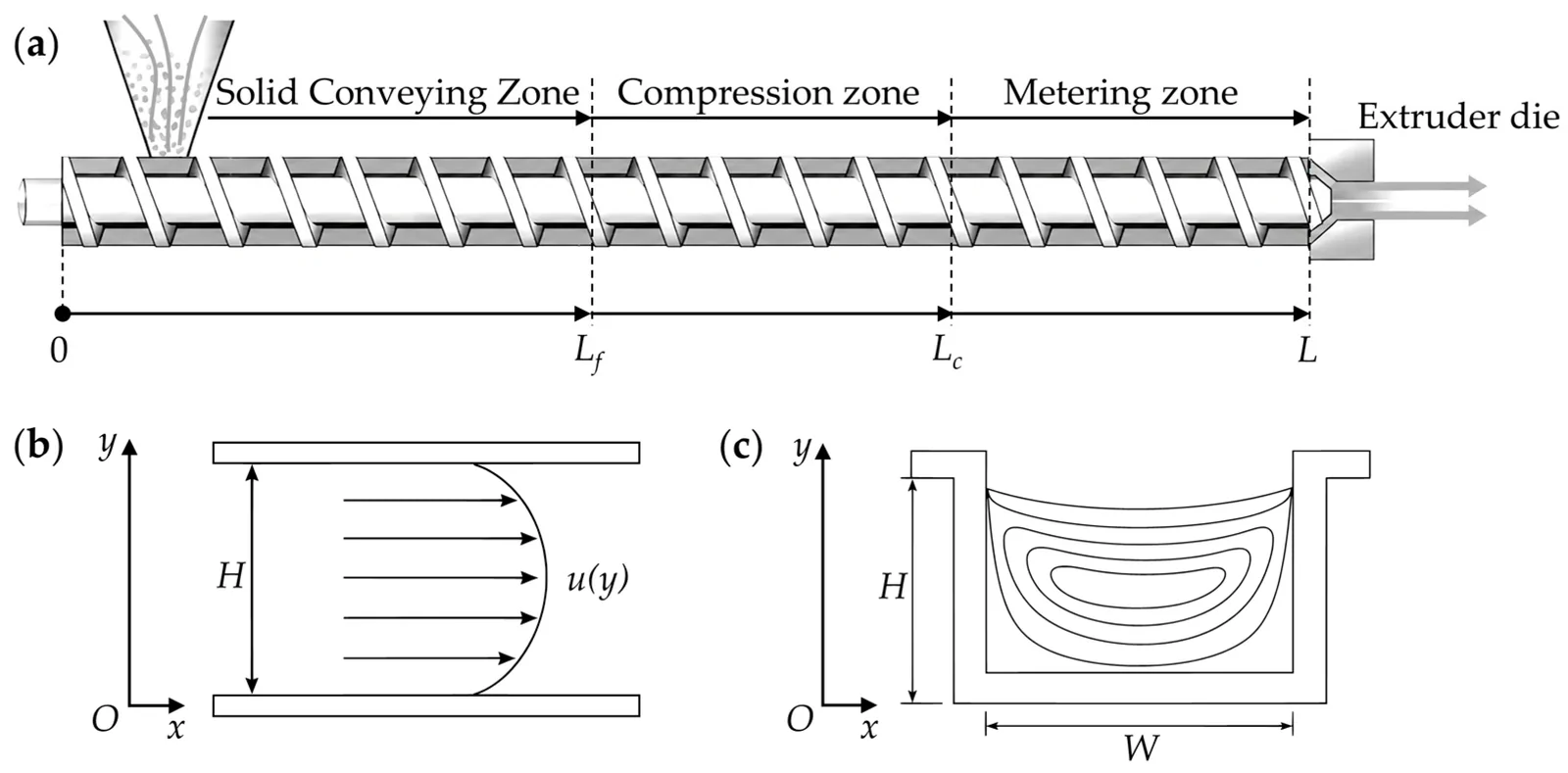
Theoretical models and analysis: (**a**) screw configuration and functional sections; (**b**) one-dimensional slit-flow approximation; and (**c**) two-dimensional channel-flow approximation. Original schematic prepared by the authors. Panels (**b**,**c**) illustrate the reduced-dimensional assumptions discussed in Section 5.2 and the associated applicability limits introduced in Section 3; they are not geometrically exact reconstructions of a specific machine.

From the mid-1980s to the 1990s, the academic community developed various analytical and semi-analytical algorithms to overcome the computational challenges of flow simulation. Among these, quasi-steady-state analysis was widely adopted as a fundamental approximation method. Based on the assumption of “axial periodic steady state,” this method simplified the transient flow field caused by screw rotation into a series of steady-state slices arranged along the helix angle [72] (Figure 6a). This simplification enabled segment-wise solution of the mass, momentum, and energy equations and provided quantitative estimates of axial pressure, flow-rate distribution, and thermal history, including in specialized configurations such as pin-barrel extruders [87,88]. These analyses also supplied a reference basis for subsequent screw-optimization studies [19].

Building on this, researchers introduced the “infinitely wide plate” assumption, simplifying the complex three-dimensional flow between the barrel and the screw into planar flow within a slit or trapezoidal channel, and established one-dimensional and two-dimensional flow-channel analysis frameworks (Figure 6b,c) [13,14]. Although such methods cannot accurately capture the details of secondary flows and dead zones near the boundaries due to the neglect of barrel sidewall drag effects and flow-channel curvature, they reduced computational costs significantly. These models revealed the coupling mechanisms among macroscopic process variables, providing an efficient reference benchmark for the preliminary design of screw configurations and the optimization of process windows [2,9].

From the mid-1990s to the 2000s, increased computational capacity and the broader availability of commercial CFD software made three-dimensional non-isothermal viscous-flow analysis more accessible in polymer-extrusion studies [9,11,89]. Compared with reduced-dimensional methods, CFD can represent geometric curvature, secondary flow, vortices, and stagnation regions in complex channels, including co-rotating twin-screw kneading elements [66,67]. The resulting fields remain model predictions whose reliability depends on constitutive choice, boundary conditions, mesh and time-step independence, and experimental validation.

However, CFD during this period faced unique bottlenecks: extremely high compound viscosity caused convergence difficulties, and intense shear-induced heating could lead to distortions in the prediction of local temperature fields under high extrusion rates [20]. Simultaneously, constrained by the homogeneous assumption, models struggled to analyze the real-time feedback of dynamic fragmentation and reorganization of filler agglomerates under high shear on local rheological properties. While smoothed particle hydrodynamics methods and Eulerian methods began to investigate mixing mechanisms and energy balances in twin-screw configurations [54,55], a dedicated constitutive coupling and multiscale CFD framework for highly filled, strongly nonlinear rubber systems remained in the exploratory stage.

Because effective in situ flow-field measurement techniques were unavailable, in-process rheological data were inferred from a limited number of pressure measurements, making the experimental dataset insufficient for systematic validation of continuous CFD fields [90]. At the same time, the effects of gravitational settling and cooling shrinkage were inherently subject to confounding factors in measured extrudate swell data, resulting in systematic uncertainties in the experimental reference values. This asymmetric development between prediction capabilities and experimental validation (e.g., contraction and capillary flows of filled rubbers) constituted a core bottleneck in the transition of numerical simulations to industrial applications [91] and shaped the trajectory of subsequent advances in experimental characterization.

### 5.3. Numerical Optimization of Die Design

The flow-channel structure of the extrusion die is a critical variable determining the dimensional accuracy and surface quality of the extrudate. Driven by advances in rheological theory, CFD-based die optimization design methods gradually matured in the late 1990s. By analytically determining the spatial distribution of pressure gradients, velocity fields, and wall shear stress within the die cavity, these methods effectively identified and eliminated traditional design defects such as flow asymmetry, stagnation zones, and uneven shear [10]. This represented a significant methodological advance from purely empirical trial-and-error toward an integrated approach combining numerical simulation with experimental refinement. Notable studies included the reverse design and validation of die heads for automotive rubber seals by Dai et al. [92]; the computer-aided optimization of the extrusion process for automotive rubber seals by Zheng et al. [93]; and the optimal design of extruder die head flow channels using the response surface method and a simulated annealing algorithm by Zhou et al. [94].

The optimization work during this period gradually overcame the limitations of isothermal steady-state analysis, beginning to incorporate the influence of shear history on viscosity evolution, as well as the nonlinear modulation of the macroscopic flow field by non-isothermal boundary conditions using finite volume methods [21,95]. This laid the methodological foundation for subsequent high-fidelity die simulations. The numerical shape optimization method proposed by Elgeti et al. integrated finite element analysis with optimization algorithms, systematically advancing the automated design of profile extrusion dies [68,96]. Similarly, Gonçalves et al. established a complete numerical modeling workflow for complex-cross-section profile dies, enabling the quantitative evaluation of exit velocity uniformity and geometric corrections [69], the principles of which were corroborated by experimental studies on profile swell and die geometry [97]. Other research further optimized die geometries utilizing polynomial networks and genetic algorithms [98], while advanced numerical frameworks clarified the mechanisms of viscoelastic and viscoelastoplastic die-swell flows [70,99]. Comprehensive 3D flow simulations from low to high aspect ratio slit dies further deepened the understanding of extrudate swell behavior across diverse geometries [100,101,102]. Additionally, specialized cost and performance analyses of dies, such as the Garvey die, were conducted to evaluate process efficiency under production constraints [103].

The period covered in this section—from the 1980s to the 2000s—was an important transition from predominantly empirical design toward quantitative analysis. Quasi-steady descriptions, reduced-dimensional models, three-dimensional CFD, and die-flow simulations supplied complementary levels of resolution; none eliminated the need for material characterization and experimental refinement.

### 5.4. Toward Unified Modeling Frameworks

Zone-by-zone models continued to be used while global or composite models were developed to integrate solids transport, plasticization, melt flow, pressure, temperature, and output along the screw [11]. In these frameworks, transition locations can emerge from the coupled calculation rather than being prescribed. Most global formulations were developed for thermoplastic extrusion, so their architecture is relevant to rubber but their parameters and quantitative predictions are not directly transferable.

Mechanism-specific work has also refined solids transport, pressure-dependent friction, plastication, heat transfer, partially filled conveying, and process dynamics [14,33,39,58]. Carreau–Yasuda, Cross, and viscoelastic equations can represent effects omitted by a simple power law, but any improvement must be demonstrated under matched material, geometry, boundary conditions, and validation metrics; computational tractability and accuracy cannot be inferred from model complexity alone [18,64,85,99].

Within the literature retained in this review, direct validation of a global rubber-extrusion model spanning deformable cold-feed transport, thermal and rheological evolution, die flow, and output remains limited [14,33,39,58]. Unified thermoplastic models are therefore classified here as transferable modeling architecture (E3) rather than as direct rubber-extrusion validation.

Simplified constitutive assumptions, limited experimental validation, and the computational cost of transient three-dimensional simulations constrained the predictive scope of this period [20,90,91]. These limitations subsequently motivated further work on nonlinear constitutive models, multiscale descriptions, and data-assisted computation.

## 6. Engineering-Scale CFD and Rubber-Specific Modeling (Increasing from the 2000s to 2015)

### 6.1. Innovations in Rheological Models and Theoretical Deepening

Whereas Section 5.1 introduced the historical emergence of differential viscoelastic models, this section focuses on their rubber-specific calibration, numerical implementation, and validation during Period III. In the early 21st century, research increasingly shifted toward specialized constitutive modeling for highly filled rubber systems. The presence of complex components such as carbon black caused the rheological behavior of rubber compounds to deviate significantly from that of unfilled polymers, driving rheological modeling from general empirical fitting toward more material-specific and physically informed descriptions (Table 4) [104]. The principal methodological advance was the development of mathematical models capable of accurately describing nonlinear rheological responses [105,106,107]. The Giesekus model introduces an anisotropic-mobility parameter (α) and a quadratic stress term and can represent shear-thinning and normal-stress effects. Its quantitative accuracy for rubber extrusion depends on material-specific parameter identification and validation [17,108]:

(4) $$\tau +\lambda \tau \nabla +\frac{\alpha \lambda }{\eta_{p}}\left(\tau \cdot \tau \right)=\eta_{p}\left(\nabla v+\nabla v^{T}\right)$$

The Phan-Thien–Tanner (PTT) model introduced a damping function *f*(tr τ) dependent on the trace of the stress tensor, dynamically coupling viscosity decay with chain relaxation [22,109]:

(5) $$f(\mathrm{tr}\;\tau )\tau +\lambda \tau \nabla =\eta_{p}(\nabla v+\nabla v^{T})$$

When calibrated and validated for the relevant material and flow, these models can represent contraction, shear, and extensional responses more effectively than simple generalized-Newtonian equations [110,111,112]. Their use does not by itself establish predictive accuracy outside the fitted formulation, geometry, or operating window.

**Figure 7:**
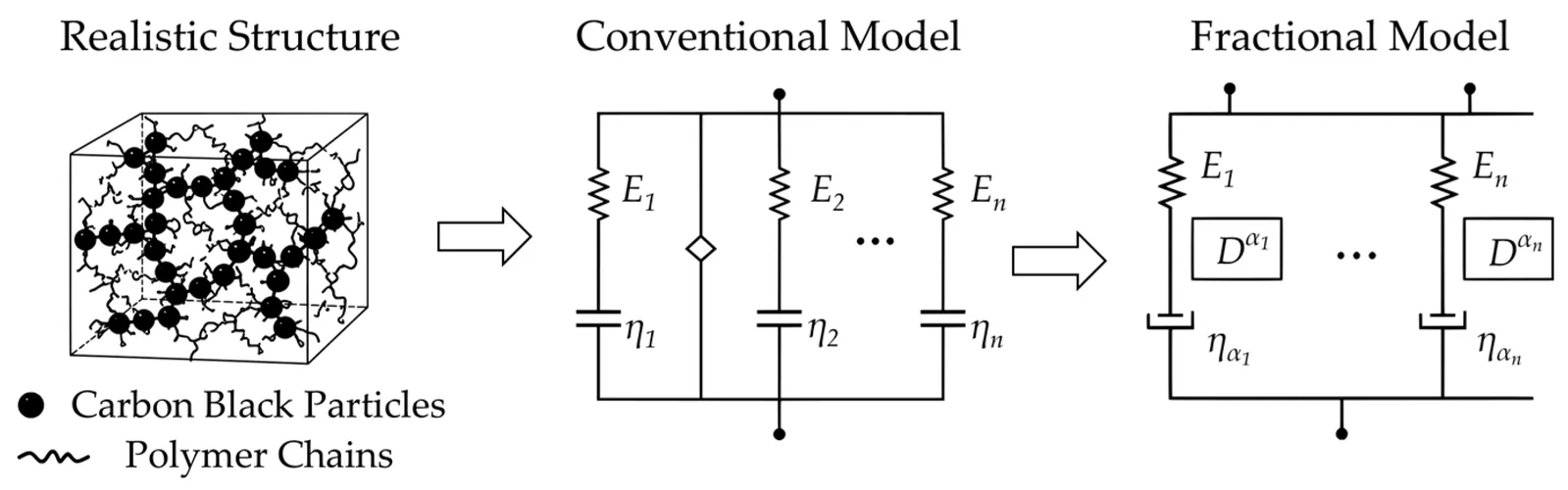
Evolution of the equivalent fractional-order microstructure model. Conceptual schematic prepared by the authors, showing hierarchical spring–dashpot analogs. The correspondence with filler-network geometry indicated here is a modeling interpretation, discussed and qualified in Section 6, and is not derived from independent structural measurement.

To characterize the long-range memory effects and structural viscoelasticity arising from filler-network reorganization within a continuum mechanics framework [113,114,115,116,117], fractional calculus theory was introduced as a modeling innovation (Figure 7) [118,119,120]. Its macroscopic stress–strain relationship is generally expressed as:

(6) $$\tau (t)+\lambda^{\alpha }D_{t}^{\alpha }\tau (t)=G\left[\gamma (t)+\lambda^{\beta }D_{t}^{\beta }\gamma (t)\right]$$

where $D_{t}^{\alpha }$ is the fractional-order derivative operator. Fitted fractional orders can provide a compact representation of broad relaxation spectra and may correlate with aspects of filler-network organization [118,121,122]. They should not, however, be interpreted as a unique or directly measured map of network fractal dimension without independent microstructural validation [123,124].

**Table 4:** Cross-period critical comparison of constitutive models, multiscale frameworks, and learned modeling approaches: (**a**) represented physical phenomena and coupling requirements; and (**b**) calibration burden, numerical robustness, computational demand, validation scale, and evidence class.

Approach or Framework	Shear-Rate Response	Elastic Response	Structural and Filler-Network Evolution	Wall-Slip and Thermal Treatment	References
(**a**) Physical capabilities and coupling requirements of constitutive models and computational frameworks.					
Power law	Shear thinning over a fitted range; low- and high-shear Newtonian plateaus not represented	Not represented	Not represented	Separate slip law; temperature dependence only through an imposed viscosity–temperature relation	[87,125]
Carreau–Yasuda/Cross	Newtonian plateaus and shear thinning over the calibrated range	Not represented	Not represented	Separate slip law; temperature dependence only through an imposed viscosity–temperature relation	[64,85]
Giesekus	Shear thinning with suitable parameters	Normal stresses; die-swell prediction when coupled with a free-surface formulation	No intrinsic thixotropy or filler-network state	Separate slip law; thermal history requires temperature-dependent parameters and non-isothermal coupling	[17,84,99]
PTT	Shear thinning; parameter- and variant-dependent	Normal and extensional stresses; die-swell prediction with a free-surface formulation; variant-dependent	No intrinsic thixotropy or filler-network state	Separate slip law; thermal history requires temperature-dependent parameters and non-isothermal coupling	[22,40,100,109]
Fractional-order	Model-dependent; fractional order alone does not guarantee shear thinning	Model-dependent; requires a suitable nonlinear tensorial formulation	History dependence represented; thixotropy and network evolution require structural coupling	Separate wall-slip and thermal formulations required	[71,118,119,120,123,124]
Heterogeneous multiscale/coupled multiscale framework	Inherited from the selected microscale model and coupling scheme	Inherited from the selected microscale model and coupling scheme	Potentially explicit when microstructural evolution is resolved	Wall and thermal effects must be represented in the microscale or coupling model	[126,127,128]
PINN	Inherited from embedded equations and training data	Inherited only when represented in the governing equations or data	Requires explicit structural variables, physics, or identifiable data	Requires corresponding boundary conditions, energy equations, or training data	[23,129,130,131,132]
Operator learning	Learned within the training distribution; not guaranteed outside it	Learned only when included in the training targets and distribution	Not physically identifiable without structural variables, constraints, or labeled data	Requires wall and thermal information in the inputs, outputs, and training data	[133,134]
(**b**) Calibration burden, numerical robustness, computational demand, and evidence base of modeling approaches and frameworks					
Power law	Steady-shear flow curve; temperature dependence for non-isothermal use	Generally robust after regularization; limited extrapolation capability	Low	Full-scale industrial dies; selected direct rubber-extrusion studies (E3), formulation-specific	[87,125]
Carreau–Yasuda/Cross	Wide-range steady-shear flow curve; temperature dependence if required	Generally robust within the calibrated range	Low	Full-scale die and screw applications; rubber-material and selected extrusion evidence (E3)	[64,85]
Giesekus	Steady-shear data plus normal-stress or transient data	High-Weissenberg-number performance is solver- and stabilization-dependent	Moderate; mesh- and solver-dependent	Laboratory dies and benchmark flows; limited direct rubber evidence and broader transferable evidence (E3)	[17,84,99]
PTT	Steady-shear data; extensional or transient data when extensional response is targeted	Solver- and parameter-dependent at high elasticity	Moderate	Benchmark flows and one experimentally validated industrial tri-screw rubber tread co-extrusion case; direct and transferable evidence (E1/E3), formulation- and geometry-specific	[22,40,100,109,135]
Fractional-order	Dynamic and transient rheology; calibration protocol is formulation-dependent	Custom implementation; dependent on memory discretization	Moderate to high	Rubber rheometry and simplified flows; mainly rubber-material evidence (E1/E2), with limited E1 validation	[71,118,119,120,123,124]
Heterogeneous multiscale/coupled multiscale framework	Microscale parameters, representative-volume-element data, and coupling assumptions	Coupling- and sampling-dependent	High to very high	Material and simplified-flow demonstrations; no full-extruder E1 validation identified in the retained corpus (E2/E4)	[126,127,128]
PINN	Collocation or training data, boundary/initial conditions, and physical parameters	Training- and formulation-dependent; extrapolation uncertain	High offline training; inference latency application-dependent	Benchmark partial-differential-equation problems and elastomer constitutive examples; no industrial rubber-extrusion validation identified in the retained corpus (E4)	[23,129,130,131,132]
Operator learning	Large multi-condition solution datasets or high-fidelity simulations	Training- and distribution-dependent	High offline training; potentially rapid inference	Benchmark equation families; no rubber-extrusion validation identified in the retained corpus (E4)	[133,134]
Neural-network surrogates/soft sensors	Large labeled process time-series datasets	Training- and domain-shift-dependent; generally stable within the training domain	Variable training cost; rapid inference	Industrial rubber-extrusion lines; selected monitoring and quality-prediction evidence (E1), task- and formulation-specific	[47,136,137,138,139]

Higher-fidelity constitutive models do not guarantee more reliable process predictions. Giesekus and PTT formulations can present convergence difficulties at high Weissenberg numbers [17,108], while fractional models add memory terms, calibration parameters, and computational demand [7,118]. Practical selection should therefore balance the physical phenomena required, parameter identifiability, numerical robustness, computational cost, and the available validation evidence.

### 6.2. Multiscale Rheological Constitutive Models

While macroscopic models such as Giesekus and PTT are highly effective for engineering predictions, their phenomenological nature cannot elucidate the microscopic mechanisms underlying nanofiller interface evolution and percolation network dynamics in highly filled systems. Between 2000 and 2015, multiscale rheological constitutive theory emerged rapidly. Its core premise is that macroscopic shear-thinning and normal stress differences are the statistical averages of microstructural processes—orientation, disentanglement, and reconstruction—within the flow field. Research followed two primary paths [17,40,123]. To avoid overlapping rankings, the model comparison is consolidated in Table 4. Part (a) identifies which phenomena are intrinsic, require an added boundary or structural model, or are learned only within a training distribution. Part (b) compares calibration burden, numerical robustness, relative cost, validation scale, and evidence class. The entries are qualitative because the underlying studies use different compounds, geometries, meshes, solvers, hardware, and metrics.

Table 4 is intentionally cross-period. It places classical constitutive equations, multiscale frameworks, and more recent learned models within a common capability-and-evidence scheme. Its placement in this section does not assign PINNs, operator-learning methods, or neural-network surrogates to Period III; these methods are discussed historically in Section 7.

Mesoscale and coarse-grained molecular-dynamics simulations have enabled direct observation of filler agglomeration, percolated particle-network effects, and polymer-chain conformations in filled polymer systems under non-equilibrium conditions [140,141,142]. Studies demonstrate that under strong shear, the filler network undergoes a dynamic “breakdown–reorganization” equilibrium, which is generally regarded as a key contributor to macroscopic transient viscosity reduction and thixotropic behavior [105,114], providing a solid physical basis for refining macroscopic constitutive equations.

The macro–micro heterogeneous multiscale coupling method embeds microscale elements as constitutive generators at selected integration points within a macroscopic computational domain and can provide local stress information without prescribing a single empirical constitutive equation [126,127]. However, its computational cost increases rapidly with the number of macroscopic integration points, microscale sampling operations, and coupling iterations [126,127,128]. In the literature retained here, applications remain concentrated in material-level or simplified-flow demonstrations, and full-scale three-dimensional rubber-extruder validation has not been established.

Although multiscale approaches remain computationally demanding and have limited full-extruder validation, they provide hypotheses linking microstructural evolution to macroscopic response and can inform reduced-order or constitutive models. Their industrial value must be assessed through formulation-specific calibration and independent validation rather than conceptual fidelity alone.

### 6.3. CFD Application and Systematic Investigation of Process Parameters

With advances in CFD methods, research expanded from isolated flow-field analyses toward broader screw-, die-, and process-parameter studies [89,143,144]. These studies investigated relationships among operating variables, predicted flow and thermal fields, and selected product-quality indicators, although full-machine rubber-extrusion validation remained limited.

Building on three-dimensional simulations, researchers systematically investigated how screw speed, barrel temperature profiles, and feed rate fluctuations govern the RTD and thermal history of rubber compounds. The results confirmed that RTD characteristics evolve highly nonlinearly: insufficient residence time compromises filler mixing uniformity, while excessive thermal exposure or shear-generated heat triggers chain scission and thermo-oxidative degradation [63,145,146].

The fundamental mechanism of scorch failure lies in the competition between RTD and Mooney scorch time. Using 3D particle-tracking simulations of EPDM flow, Launay et al. (2014) [44] found that viscous dissipation strongly accelerates local cure progression in dies. Their work across straight, bend, and bifurcation geometries demonstrates that optimizing die design can effectively control scorch onset and improve cure uniformity.

**Figure 8:**
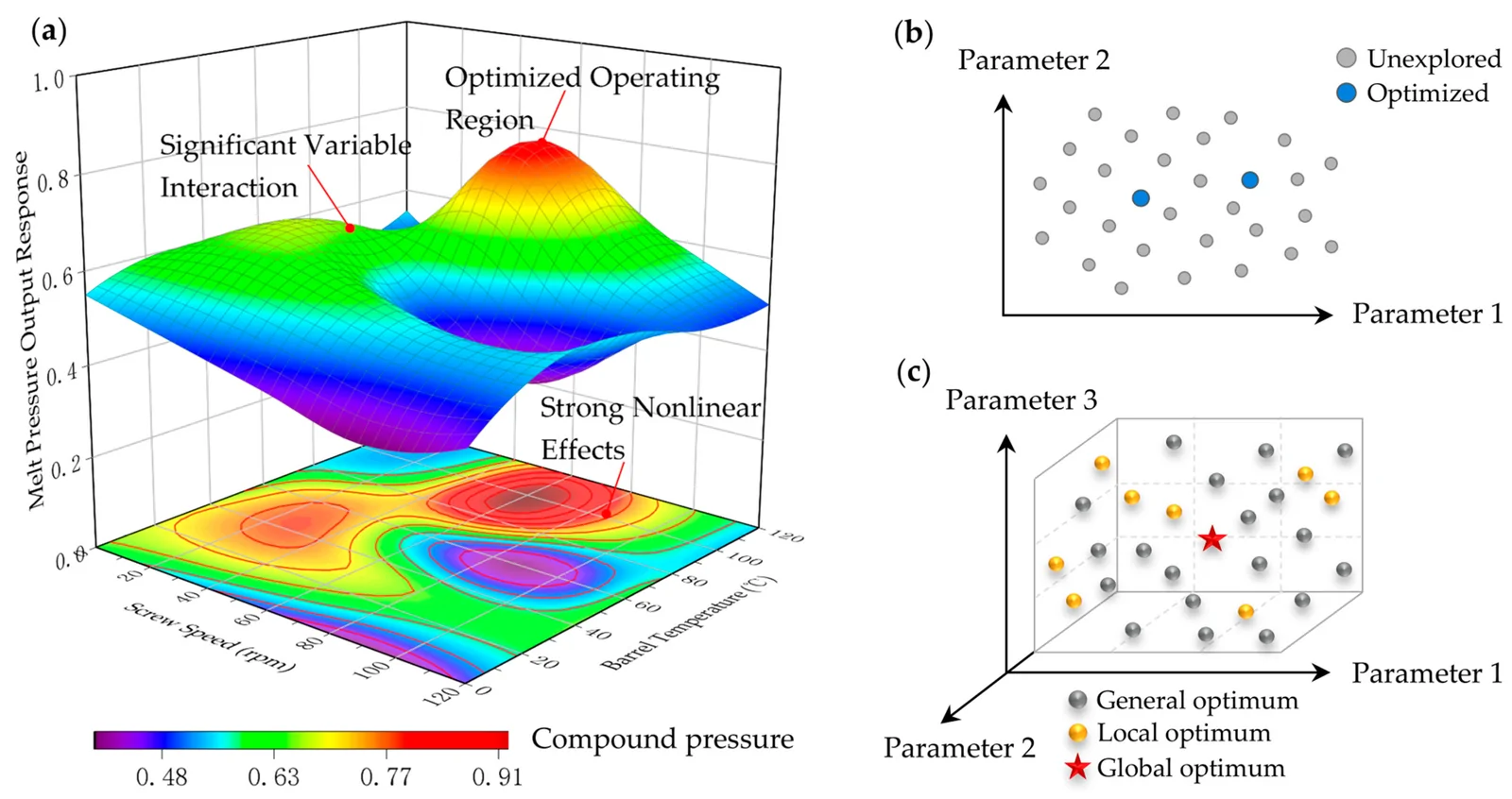
Qualitative illustration of coupled process-parameter effects: (**a**) interactions among screw speed, barrel temperature, and feed rate; (**b**) a single tested combination; and (**c**) a multi-dimensional search space. Original schematic prepared by the authors. Nominal axis values are illustrative and do not correspond to a measured or simulated dataset.

As high-filler tire formulations became widespread, interactions among screw speed, temperature, pressure, feed rate, residence time, and formulation became increasingly important (Figure 8a) [4,108,147]. These observations show why one-factor linear extrapolation may fail outside a tested range and motivate multivariable experiments, coupled models, and control methods (Figure 8b,c) [148,149]; they do not establish a universal process surface across machines or compounds.

### 6.4. Screw Configuration Optimization and Mixing Efficiency Enhancement

Screw topology is a primary determinant of twin-screw extruder performance in processing high-viscosity, highly filled rubber systems [55]. Modern screws comprise modular functional units—conveying sections, shearing/mixing sections, and degassing sections—whose geometric parameters and assembly sequence directly govern the melt’s thermomechanical history, thereby determining mixing efficiency and product quality [150].

CFD-based parametric studies indicate that kneading-block length, pitch, channel depth, clearance, and disc offset can influence distributive and dispersive mixing, residence-time distribution, pressure, and specific energy consumption [55,56,150,151]. Reported improvements are specific to the simulated geometry, formulation, constitutive assumptions, and operating window and require experimental confirmation before transfer to production equipment.

Addressing the nonlinear friction characteristics of cold-feed rubber, researchers recently introduced pressure-dependent friction constitutive models into quantitative analysis of the screw feeding section [57]. Adjusting the feeding section geometry—groove depth, helix angle, and groove bottom roughness—enables active regulation of the $f_{b}/f_{s}$ ratio within specific pressure ranges, broadening the stable conveying window [31,34]. Notably, a mismatch between feed rate and screw speed generates localized adhesion dead zones at the interface of cold-feed rubber, triggering intermittent feeding fluctuations that propagate to the compression zone and affect metering-zone outlet-pressure stability [30,58]. This cross-section coupling effect is a distinctive feature of the three-zone theory in rubber systems compared to thermoplastics and represents a critical physical constraint for future digital design of cold-feed screws.

Addressing the complex dispersion challenges in filled systems, experimental and modeling studies on the breakup of silica and carbon-black agglomerates and the visco-elastic-plastic properties of filled natural rubber further refined element geometry design [152,153]. Viewed across Period III, progress was increasingly equipment- and mechanism-specific rather than a universal extension of the three-zone theory. In conventional cold-feed single-screw extrusion, pressure-dependent friction and deformable-feed analyses improved the treatment of feeding stability. In twin-screw systems, element geometry, residence-time distribution, elongational deformation, and dispersive and distributive mixing indices became more appropriate descriptors than direct zone-by-zone analogies. At the die and downstream stages, rubber-specific constitutive models and CFD enabled more detailed analysis of viscoelastic deformation, thermal history, scorch risk, and dimensional stability [58,63,120].

Nevertheless, substantial gaps remained between idealized models and industrial operation. Thermo-oxidative degradation, filler-network evolution, wall slip, formulation variability, and transient non-equilibrium effects were commonly simplified or represented through effective parameters. These limitations motivated the transition toward more strongly coupled multiphysics models, cross-scale descriptions, online sensing, and physics-informed or data-assisted methods.

## 7. Multiphysics, Sensing, and Data-Enabled Methods (Increasing After 2015)

Since 2015, rubber-extrusion research has increasingly considered multiphysics coupling, online sensing, reduced-order modeling, and data-enabled optimization alongside established empirical and numerical methods. This section reviews the evidence for these approaches and distinguishes demonstrated component technologies from proposed full-process intelligent systems.

### 7.1. Multiphysics Coupled Simulation: Advancing the Fidelity of Physical Mechanism Modeling

**Figure 9:**
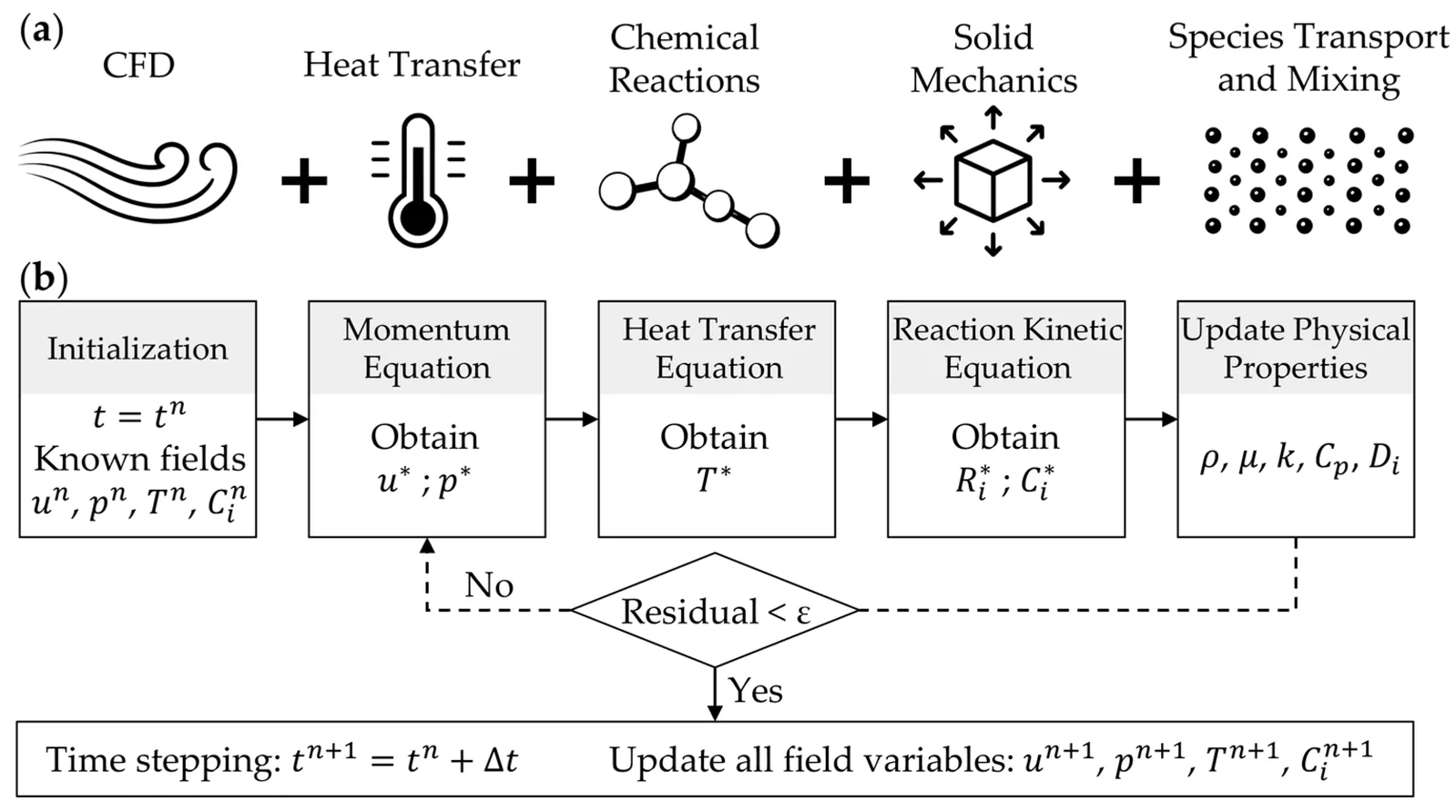
Multiphysics coupling theory: (**a**) schematic of multiphysics coupling; (**b**) flowchart of multiphysics coupling solution. Original schematic prepared by the authors, summarizing the coupling structure and solution sequence described in the cited literature; it does not represent a specific implementation or its results.

Research during this period has extended single-field simulations by coupling flow, heat transfer, and, where relevant, reaction kinetics [24,59,135,146]. Such models can represent interactions omitted by simpler analyses, but their accuracy remains bounded by constitutive assumptions, parameter calibration, boundary conditions, numerical convergence, and the availability of rubber-specific validation data (Figure 9).

With respect to coupled heat transfer and flow dynamics, recent work has emphasized viscous dissipation and temperature–flow feedback [108]. Because rubber compounds have low thermal conductivity and high viscosity, shear-induced heat generation should be represented when predicting local temperature gradients near abrupt geometry changes [40,108]. For highly filled systems, wall slip, filler-network evolution, and interfacial effects are often represented through effective parameters or simplified multiphase descriptions [60]; these are macroscopic approximations and do not resolve the actual filler microstructure.

Multiphysics models can also incorporate viscoelastic stress-history effects and reproduce phenomena such as die swell under calibrated conditions [71,154]. Their computational cost generally confines high-fidelity models to offline analysis. Reduced-order and surrogate models may support faster estimation or monitoring [25,64,155,156], but online closed-loop use requires reported latency, uncertainty, stability, and validation under disturbances and formulation changes.

**Figure 10:**
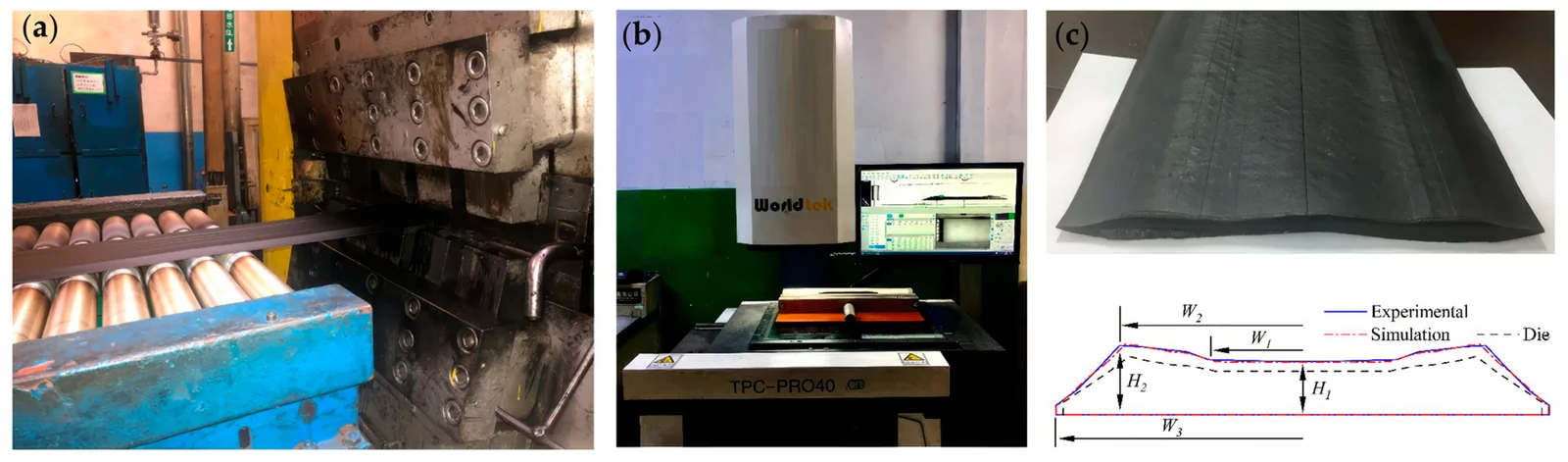
Representative experimental validation of tri-composite tire-tread co-extrusion: (**a**) industrial tri-screw co-extrusion equipment; (**b**) extruded tread sample and cross-section measurement; and (**c**) comparison between the simulated and experimentally measured tread profiles. Adapted from Wang et al. [135]. under the Creative Commons Attribution 4.0 license.

A representative example of direct rubber-extrusion validation was reported by Wang et al. [135] for the tri-composite co-extrusion of tire tread. Three rubber compounds were characterized experimentally, fitted using the PTT constitutive model, and subsequently implemented in a three-dimensional viscoelastic free-surface simulation. The predicted tread profile was compared with the cross-section measured from an operating tri-screw extrusion line, as illustrated in Figure 10.

The reported dimensional deviations between the simulated and measured tread profiles were below 6%, demonstrating that a calibrated viscoelastic model can provide useful engineering predictions of die swell and compound-interface deformation. Nevertheless, the validation was conducted for a specific extruder head, compound set, and operating condition. The results therefore constitute direct but formulation- and geometry-specific evidence rather than validation of universal transferability across rubber-extrusion systems [135].

### 7.2. Digital-Twin Concepts and Applications

**Figure 11:**
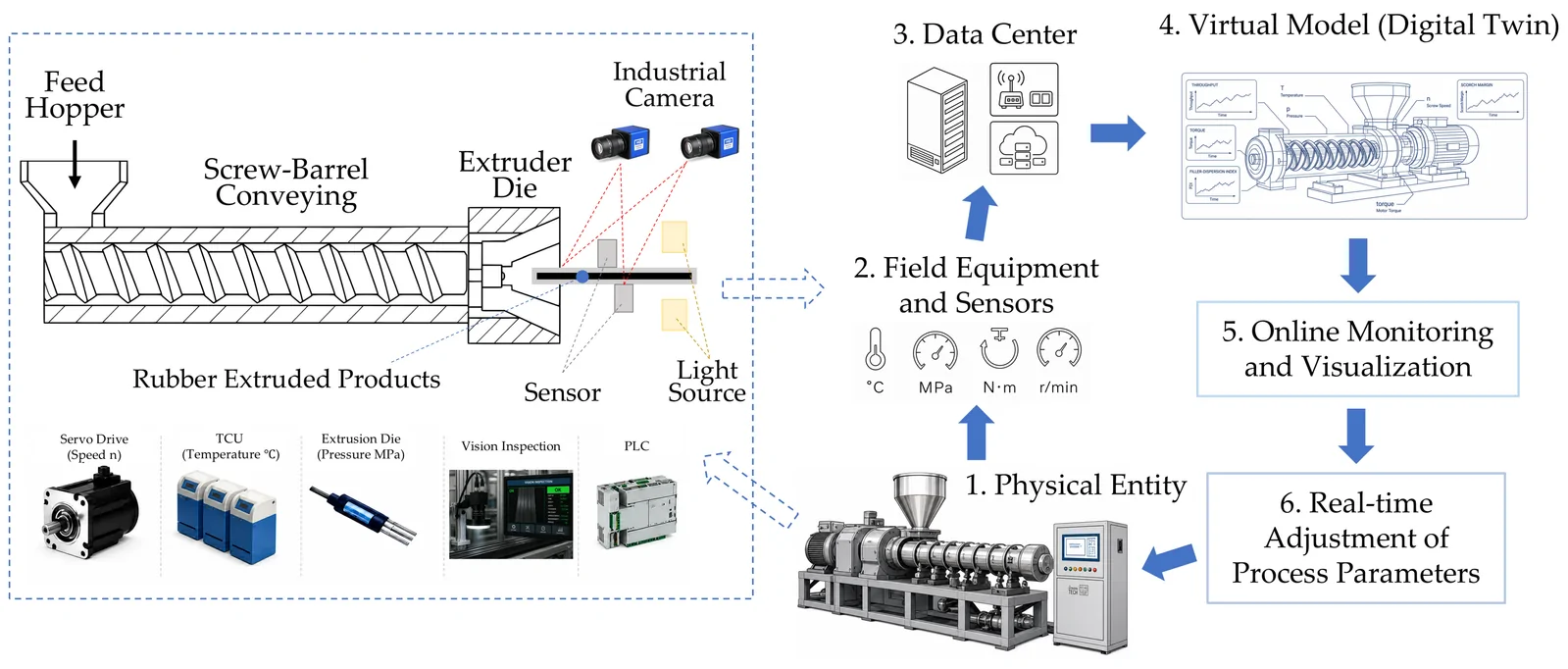
Schematic of the digital-twin architecture. Original schematic prepared by the authors from the architectures discussed in [25,155,157]. The diagram does not represent a deployed system and does not establish online prediction or control performance.

A digital twin is generally understood as a virtual representation that is updated with measurements from a physical asset and supports monitoring, diagnosis, prediction, or decision-making (Figure 11) [157]. For rubber extrusion, a credible implementation would need to integrate variables such as screw speed, torque, barrel temperature, pressure, and throughput with material-specific state estimates. Potential benefits include more efficient commissioning and improved consistency, but these benefits depend on model updating, data quality, validation, and the level of bidirectional integration actually demonstrated [3,158].

Transferable studies on general polymer single-screw extrusion have developed dynamic and surrogate models for melt conveying and process-state prediction [64,159]. However, these models do not directly represent filler-network evolution, reactive silica–silane coupling, wall slip, or scorch in rubber extrusion. A rubber-specific digital twin would therefore require additional material-state variables, potentially including a filler-dispersion index, reaction-progress variables for silica–silane systems, and a scorch-safety margin based on residence and scorch times. At present, these quantities should be regarded as candidate state variables or inferred indicators rather than universally validated online measurements [35,44,80]. Their definition, observability, and uncertainty must be established for the relevant formulation and equipment before they are used for closed-loop decisions.

Direct industrial evidence for complete rubber-extrusion digital twins remains limited. The cited literature supports components such as temperature control, soft sensing, process modeling, and data integration, but it does not collectively demonstrate a universally validated lifecycle twin for tire compounds or automotive seals. Claims concerning reconstruction of internal viscosity, scorch prediction, or devulcanization state should therefore be treated as application targets unless supported by independent plant-scale validation and reported error, latency, and robustness metrics [25,26,44,90,160].

A transferable demonstration in plastic stratified co-extrusion showed that a digital process twin can integrate screw characteristics, die-flow models and sensor data to support operating-point evaluation [158]. Its transfer to industrial rubber extrusion requires additional treatment of filler-network evolution, wall slip and scorch. When the twin model detects process deviation or quality fluctuation, generating optimal control strategies from extensive historical and real-time data has become the key bottleneck constraining further evolution toward intelligent manufacturing. This challenge has driven the convergence of data-driven methods and intelligent control technologies [3]. Soft-sensor and data-enabled process control components have been demonstrated for rubber extrusion [25], while life-cycle assessment and life-cycle costing have been applied to the eco-design of rubber products [161]. Their integration within a full digital-twin framework remains a proposed research direction.

Nevertheless, key scientific challenges remain in constructing full-process digital twins, integrating heterogeneous data streams in a physically interpretable manner, and enabling adaptive decision-making under extreme operating conditions.

### 7.3. Data-Driven Methods and Intelligent Control: Evidence and Validation

**Figure 12:**
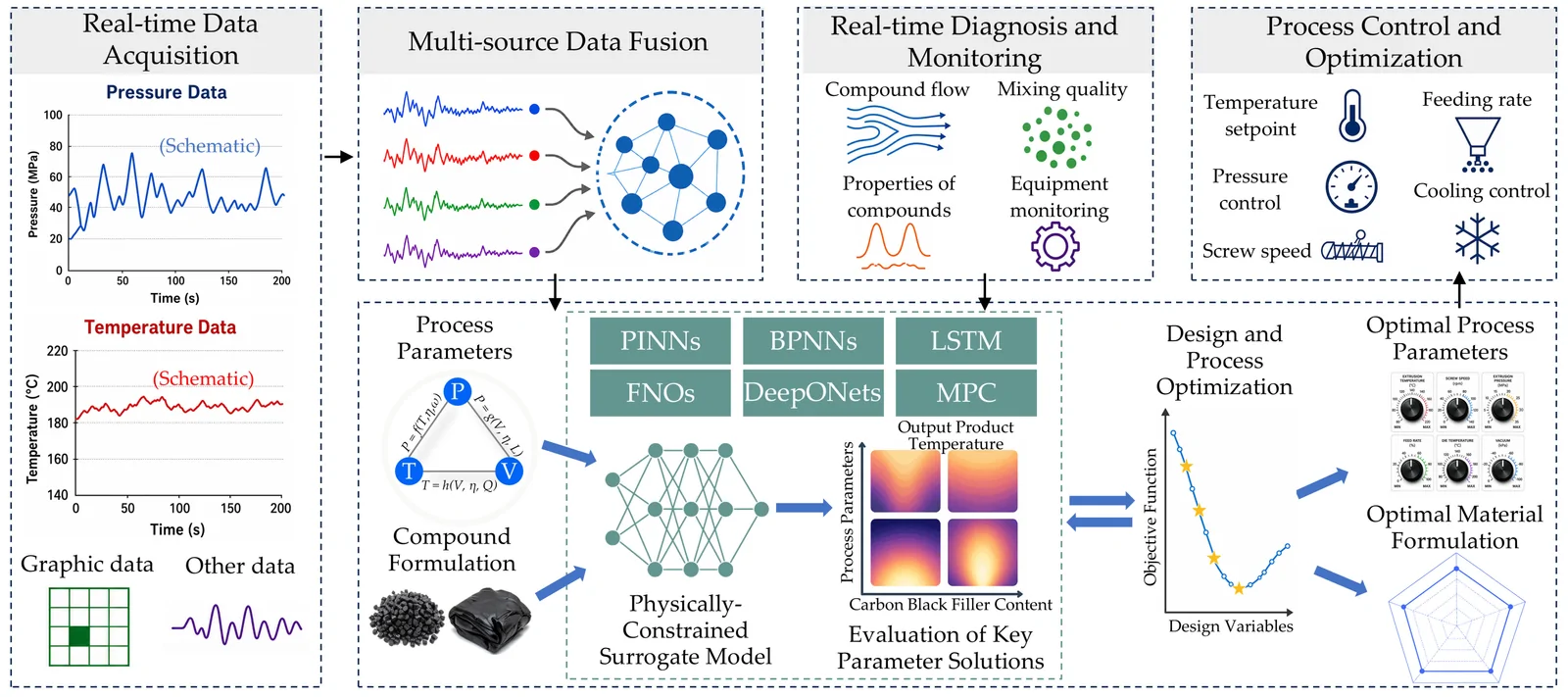
Conceptual workflow for online sensing, diagnosis, model updating, and process control. Original schematic prepared by the authors. The diagram reports no measured signals and does not establish industrial validation.

Traditional optimization of rubber-extrusion process parameters primarily relies on orthogonal experiments and response surface methods, which struggle to effectively address high-dimensional nonlinear optimization problems [2,94]. The dynamics of filler-network fragmentation and reorganization, the coupled temperature- and shear-dependence of viscosity, and the competition between residence time and scorch time create a high-dimensional nonlinear problem that classical statistical methods cannot adequately resolve. The introduction of neural-network and other data-driven models offers new possibilities for addressing this challenge (Figure 12) [136,137].

One emerging route for embedding physical constraints in data-driven models is physics-informed machine learning [130,132]. In principle, conservation laws, constitutive relations, and cure kinetics can be incorporated into the learning objective to estimate temperature, shear-rate, or residence-time fields. However, the cited studies do not yet establish real-time, closed-loop PINN control of an industrial rubber extruder. Training cost, optimization stiffness, sparse internal measurements, changing formulations, and inference latency remain practical limitations; rubber-specific validation should precede claims of operational readiness.

Data-driven studies relevant to rubber processing demonstrate component capabilities rather than a fully integrated intelligent extrusion line. Time-series models, neural networks, spectroscopy, and soft sensors have been investigated for defect detection, viscosity or swell prediction, mixing optimization, and material-state estimation [47,64,138,139,162,163,164,165]. Performance is typically formulation-, machine-, and dataset-dependent. Future reports should therefore include independent test sets, uncertainty, sampling and inference latency, robustness to disturbances, and cross-formulation or cross-machine evaluation.

Candidate sensing architectures can combine pressure, temperature, torque, imaging, and spectroscopic signals [25,27,90]. State-estimation and control methods, including Kalman and particle filtering, neural networks, fuzzy proportional–integral–derivative (PID) control, and model-predictive control, have been investigated for estimating material states and adjusting process variables [26,166,167,168,169,170]. These studies demonstrate relevant components, but evidence for end-to-end closed-loop operation across an entire industrial rubber-extrusion line remains limited. Reports should distinguish measured variables from inferred states and provide calibration drift, sampling rate, latency, stability, and independent validation results.

**Figure 13:**
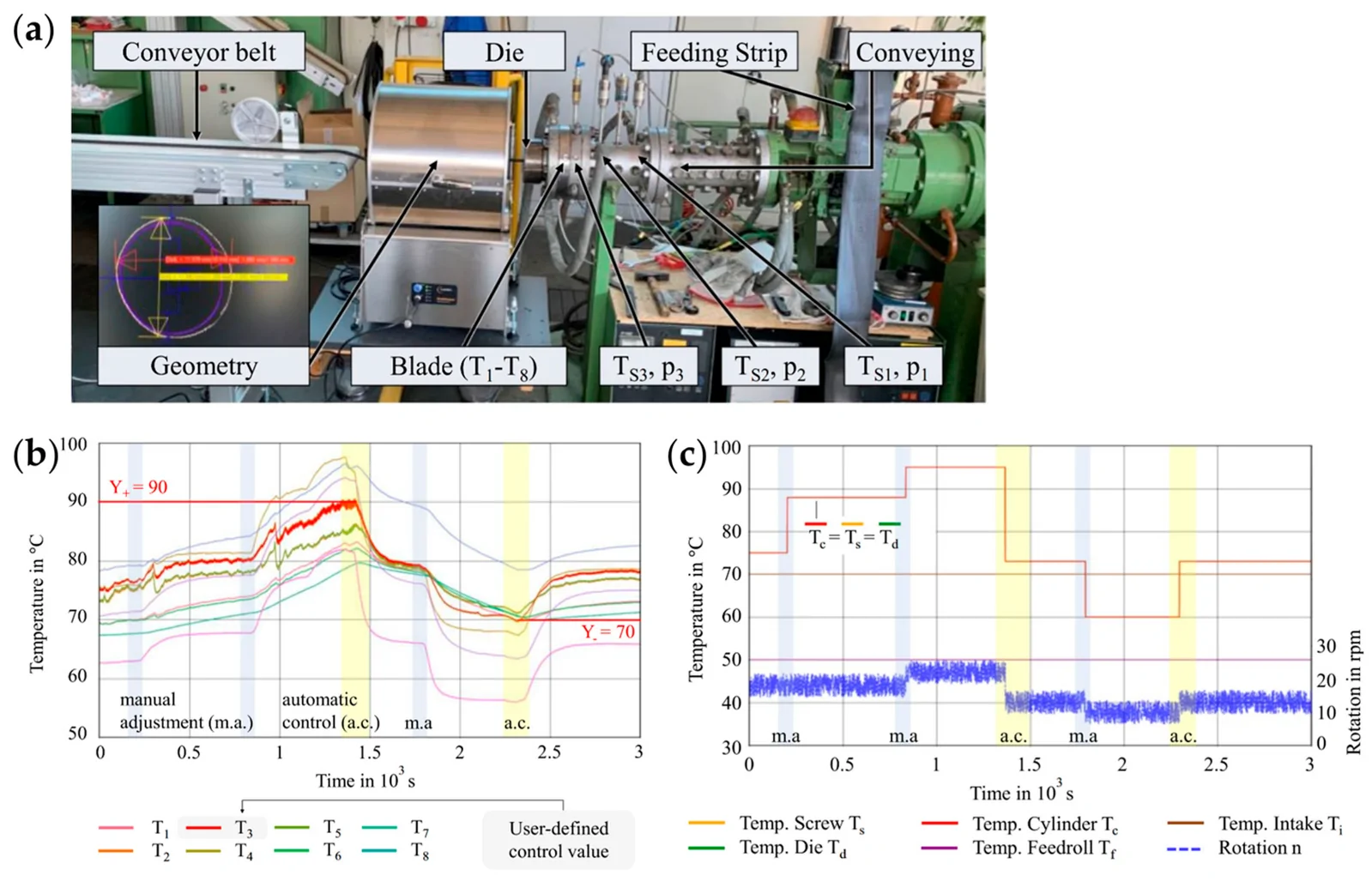
Inline experimental validation of data-enabled temperature control in an EPDM rubber-extrusion line: (**a**) extrusion equipment and locations of temperature, pressure, and optical measurements; (**b**) measured process-output responses during the control experiment; and (**c**) corresponding changes in screw speed and temperature-control inputs. The light-blue and light-yellow shaded bands indicate manual adjustment events (m.a.) and automatic control interventions (a.c.), respectively. Adapted from Lukas et al. [26] under the Creative Commons Attribution 4.0 license.

Direct line-level evidence has been reported for data-enabled temperature control in EPDM rubber extrusion. Lukas et al. [26] instrumented an operating extrusion line with multiple compound-temperature and pressure sensors, a multi-point temperature blade at the screw-channel outlet, and optical measurement of the extrudate geometry. The resulting process data were used to train and validate an artificial-neural-network-based prediction and control system, as summarized in Figure 13.

The model was developed using 14,923 measurement points for each monitored temperature and achieved an average mean absolute percentage error (MAPE) of 0.68% and a mean squared error (MSE) of 0.63 °C^2^. During inline validation, control interventions were transmitted within approximately 44–68 ms, and the controlled temperature returned to the prescribed target with a deviation below 1 °C [26]. This study therefore provides E1 evidence for a component-level, data-enabled control function on an operating rubber-extrusion line. However, the validation remains specific to the investigated EPDM compounds, sensor configuration, and production line and does not establish autonomous control of the complete extrusion process.

Existing data-enabled methods face common limitations: physics-informed models can be difficult to train, purely data-driven models are sensitive to domain shift, and rubber extrusion lacks standardized benchmark datasets [1,23,129]. Operator-learning methods such as Fourier neural operators (FNOs) and Deep Operator Networks (DeepONets) can provide rapid surrogate predictions after training, but their accuracy is bounded by the training distribution, discretization, and representation of highly nonlinear viscoelastic behavior. Reported speedups or zero-shot performance in other problems governed by partial differential equations should not be transferred directly to industrial rubber extrusion without rubber-specific validation [131,133,134,171,172]. Data-driven and generative models have also been explored for thermoplastic-vulcanizate formulation design, but this constitutes material-design evidence rather than extrusion-line validation [173].

Even for a nominally unchanged formulation, storage history, ambient conditions, and batch-to-batch variability should be treated as potential sources of variation in the initial material state [164]. Intelligent models should therefore represent the joint formulation–history–operating-condition state rather than relying exclusively on regression against nominal process setpoints. This requirement becomes particularly important when new low-rolling-resistance or reclaimed-rubber formulations introduce additional material variability and sustainability constraints.

### 7.4. Application Drivers and Sustainability: Electric-Vehicle Tires, Reclaimed Rubber, and Circular Processing

Beyond advances in modeling, sensing, and control, the recent development of rubber extrusion is increasingly shaped by application-level requirements for lower rolling resistance, improved resource efficiency, reclaimed-material processing, and measurable environmental performance. Electric-vehicle tire applications and circular-manufacturing objectives are driving increased interest in highly filled, low-rolling-resistance compounds, reclaimed rubber, and more resource-efficient extrusion processes [161,174,175,176,177]. These application pressures add formulation variability, reaction kinetics, thermal-history control, material recovery, and sustainability constraints to the conventional objectives of throughput, dimensional stability, and surface quality.

Silica-filled SBR and related tire-tread compounds exhibit filler-network evolution and strongly formulation-dependent rheological behavior [77,78,113]. Where silane chemistry is involved, reaction and thermal-history effects should be represented separately and validated for the relevant formulation [59,178]. Feed stability, segmented temperature control, and, where feasible, rheological or spectroscopic monitoring are relevant process considerations, but their integrated industrial performance should be demonstrated rather than assumed.

In green circular-economy applications, continuous devulcanization of waste rubber involves coupled thermal, mechanical, and chemical constraints and requires stable feeding and controlled thermal history [160,179]. Reclaimed and devulcanized rubber materials may introduce additional formulation and batch variability [174,175,176,177]. These developments motivate evaluation of energy use, material recovery, emissions, and product-property retention together with throughput and dimensional quality; they do not yet define a single standardized intelligent-processing paradigm.

Viewed through the bounded three-zone theory, recent methods can support coordinated monitoring of feeding, thermomechanical conditioning, metering, and downstream die behavior [26,166,167,168,169,180]. Sensing, digital twins, and control remain system-level layers rather than new screw zones, and current evidence supports targeted applications more strongly than full-process autonomous coordination.

## 8. Conclusions and Outlook

### 8.1. Critical Synthesis and Main Conclusions

Rubber extrusion has developed through overlapping changes in equipment, rheological characterization, numerical modeling, sensing, and control (Table 5). Each increase in model resolution introduces additional requirements for parameters, computation, and validation. The continuing engineering challenge is therefore not the pursuit of maximum model complexity but the selection of the least complex model that captures the mechanisms required for a stated decision and is supported by appropriate evidence.

The periods indicate changes in dominant research emphasis rather than discrete technological stages. The boundaries are approximate, and methods associated with different periods continue to overlap and coexist. Representative advances include both direct rubber-extrusion evidence and, where indicated, enabling evidence transferred from adjacent domains.

When the four analytical periods are viewed through the bounded three-zone theory, persistent limitations become clearer. Early analytical models supported conveying and pressure estimates but simplified material behavior; CFD resolved more geometric detail but often retained homogeneous constitutive assumptions; multiscale models proposed links to filler-network dynamics but remained computationally demanding; and recent sensing or data-enabled methods introduced new requirements for datasets, validation, latency, and transferability. These approaches continue to coexist, and none should be treated as a universal replacement for the others.

Overall, the bounded three-zone theory remains useful for selected physical questions in conventional single-screw and cold-feed rubber extrusion, but it should not be extended uncritically to twin-screw elements, dies, or system-level sensing and control. Across the four analytical periods, the principal unresolved issue is not simply insufficient model complexity, but incomplete validation of the coupled relationships among filler-network evolution, wall slip, thermal history, and macroscopic flow. Progress therefore depends on transparent datasets, matched material and process characterization, and validation across formulations, machines, and operating windows.

### 8.2. Current Industrial Challenges and Evidence Limitations

Despite significant progress in rheological modeling and process monitoring, the intelligent transformation of rubber extrusion remains constrained by four major industrial and technological bottlenecks. Interpretation and comparison of the reported progress are additionally limited by heterogeneity in the available evidence and by limitations of the retained review corpus.

First, three-dimensional non-isothermal CFD can be too computationally demanding for direct use at control-relevant latency. Because models are usually calibrated for particular compounds and geometries, predictive accuracy may decline when they are transferred to highly filled, multicomponent formulations or operating ranges not represented during calibration [60,108].

Second, many production sensors are mounted on the barrel or at a limited number of downstream locations. Thermal inertia, conduction delay, fouling, and sparse spatial coverage can limit reconstruction of transient conditions in the melt core and can introduce lag into feedback control [26,166].

Third, the effectiveness of data-driven algorithms relies heavily on high-dimensional, high-quality operational time-series data. To date, no standardized process atlas database has been established in this field, and cross-machine and cross-formulation transfer learning remains nascent. Consequently, pre-trained models face significant domain shift challenges when deployed on production lines [25,138,163].

Fourth, current optimization studies primarily focus on isolated quality metrics such as geometric dimensional accuracy or surface defects, lacking systematic integration of energy intensity, carbon footprint, and reclaimed-rubber recycling rates. The development of eco-design frameworks that simultaneously account for life-cycle assessment and life-cycle costing remains essential for sustainable rubber manufacturing [161]. Moreover, while advances in functionalized filler systems have demonstrated significant improvements in tire tread compound performance [77,78], these formulations introduce additional complexity in extrusion process control that current optimization frameworks have yet to fully address.

The evidence base is heterogeneous in compound formulation, equipment, scale, operating window, constitutive model, instrumentation, and reported metric. Direct rubber-extrusion validation is less common than rubber-material studies or evidence transferred from thermoplastic extrusion and generic computational research. These differences prevent meaningful pooled performance estimates and limit cross-study rankings.

The cited corpus was assembled iteratively and is not claimed to be exhaustive. A complete record-level search log was not retained, industrial datasets are underrepresented, and older studies often omit information required for reproducibility. The conclusions and roadmap should therefore be interpreted as an evidence-graded critical synthesis rather than a formal meta-analysis.

### 8.3. Future Research Priorities and Roadmap

In light of these bottlenecks, future research should prioritize five mutually dependent directions: transparent benchmark datasets, minimum validation and uncertainty-reporting protocols, mechanism–data integration, transferable sensing and control, and measurable sustainability assessment. Progress should be coordinated across theoretical, technological, and system-level dimensions rather than evaluated through isolated improvements in model complexity or prediction accuracy.

At the theoretical level, combining mechanistic models with data-driven methods may improve physical consistency and reduce data requirements. PINNs, reduced-order models, and neural operators are relevant candidates [133,134], but their value should be demonstrated through rubber-specific benchmarks that report error, uncertainty, computational latency, and performance under formulation and operating-condition changes. Dynamic process-window models should likewise be calibrated against measured material variability and equipment constraints.

On the technology front, combinations of pressure, temperature, torque, imaging, ultrasound, and near-infrared spectroscopy could improve estimation of internal process and material states. Candidate sensing architectures should be assessed for sampling rate, calibration drift, fouling, maintenance, and uncertainty. Reduced-order models and model-predictive control may support faster coordinated control, but claims of millisecond-level operation require end-to-end latency and closed-loop stability evidence.

At the system level, future studies should move from single-machine demonstrations toward reproducible evaluation across equipment, formulations, and production campaigns. Multi-objective optimization should report quality, throughput, energy use, material loss, and emissions, while transfer-learning or meta-learning methods should be evaluated with predefined cross-domain tests. Reclaimed-rubber applications should additionally report property retention and environmental metrics [174,175,176,177].

Near-term work should establish shared reporting templates and open benchmark datasets linking formulation, rheology, equipment geometry, operating conditions, sensor signals, product quality, and uncertainty. CFD and data-driven studies should report independent validation, mesh or model convergence, calibration ranges, inference latency, and failure cases.

Medium-term work should test wall slip, filler-network evolution, reduced-order models, and multimodal soft sensors across formulations and machines. Cross-domain evaluation should be predefined rather than demonstrated only after retraining, and control studies should report closed-loop stability and end-to-end latency under disturbances.

Longer-term integration should be judged by measurable outcomes: dimensional and surface-quality consistency, scorch safety, throughput, specific energy use, material loss, reclaimed-rubber content, emissions, and property retention. These are proposed validation priorities, not established industry benchmarks.

The bottlenecks are neither equally tractable nor independent. The highest-priority actions are to establish enabling infrastructure: a traceable benchmark dataset and a minimum validation protocol are preconditions for uncertainty quantification, cross-study comparison, and the safe deployment of reduced-order or learned models. Table 6 presents proposed deliverables and validation criteria; its thresholds are research targets to be justified for each process, not established industry standards.

The horizons are indicative planning intervals rather than forecasts or fixed deadlines. The evaluation criteria are proposed research and reporting targets, not established industry standards. Thresholds should be justified for the relevant compound, equipment, operating window, and decision context.

Reduced-order models, multimodal sensing, physics-informed learning, and neural operators may help address the trade-offs among model fidelity, transferability, and inference speed. Their contribution to intelligent and sustainable rubber extrusion will depend on uncertainty-aware, plant-relevant validation and on measurable improvements in quality, resource use, and process robustness.

## Figures and Tables

**Table 1:** Positioning of the present review relative to closely related prior reviews. Compiled by the authors.

Review	Material System and Scope	Rheology and Models	Data and Control	Historical Structure	References
Gorakifard et al.	Tire-extrusion literature; tooling, rheology, equipment, computer-aided engineering, and monitoring/control	Thematic mapping; no model-by-model comparison	Monitoring/control mapped; no rubber-specific validation hierarchy	Bibliometric and thematic evolution	[1]
Gaspar-Cunha et al.	General polymer processing, mainly thermoplastics; screw and die optimization	Models used as optimization inputs; no systematic model comparison	Multi-objective and evolutionary optimization; limited online control	No explicit periodization	[2]
Hopmann et al.	Uncured tire-tread rubber; rheometry, die flow, swell, melt fracture, wall slip, and scorch	Detailed rubber rheology and characterization	Data-driven methods and online control not systematically reviewed	No explicit periodization	[4]
Hyvärinen et al.	General polymers and composites; extrusion process, machine, screw, and die modeling	General polymer-flow models; no rubber-specific comparison	Limited treatment	No explicit periodization	[9]
Pittman	Thermoplastic and rubber profile extrusion; profile-die and post-die optimization	Rheology, wall slip, instabilities, swell, residence time, and thermal effects	Computer-aided die optimization; no online-control focus	No explicit periodization	[10]
Lewandowski and Wilczyński	General polymers; twin-screw transport, plasticization, melt flow, CFD, optimization, and scale-up	Generalized-Newtonian and viscoelastic models	Optimization and scale-up; online control not a primary focus	No explicit periodization	[11]
This review	Highly filled, highly viscoelastic rubber; conveying, thermomechanical conditioning, metering, die flow, sensing, and control	Generalized-Newtonian, viscoelastic, fractional, multiscale, and learned models compared by capability and evidence	Digital twins, physics-informed neural networks (PINNs), sensing, and control assessed; rubber-specific evidence distinguished from transferable evidence	Four overlapping, criterion-based periods	-

**Table 2:** Indicators anchoring the period boundaries adopted in this review. Compiled by the authors from the cited sources.

Approximate Boundary	Constitutive Modeling	Computational Capability	Sensing and Control Maturity	References
~1980s (working marker)	Increasing use of power-law and other generalized-Newtonian approximations in extrusion analysis	One- and two-dimensional numerical channel-flow analyses increasingly reported	Conventional temperature and pressure measurements; predominantly local or manual control	[13,14,15,16]
~2000s (working marker)	Differential viscoelastic models more widely studied; rubber-specific applications remained uneven	Engineering-scale three-dimensional CFD increasingly accessible	Inline pressure and temperature measurement and standardized rheometry more widely reported; recipe and supervisory control increasingly used	[17,18,19,20,21,22]
~2015 (working marker)	Fractional, structural, and multiscale approaches became more visible in rubber and adjacent fields	graphics processing unit (GPU)-accelerated computing, reduced-order models, and learned surrogates became increasingly feasible; online latency remained application-dependent	Multimodal sensing and digital-twin or learning-based control demonstrated in selected cases; direct rubber-extrusion validation remained limited	[23,24,25,26,27]

**Table 3:** Applicability of the three-zone theory across functional regions and analytical periods.

Functional Region or Element	Period I: To 1980s	Period II: 1980s–2000s	Period III: 2000s–2015	Period IV: 2015–Present	References
Single-screw cold-feed conveying—Conditionally applicable; rigid-plug and Coulomb-friction assumptions require modification	Macroscopic kinematics and Coulomb friction	One-dimensional pressure–throughput models	Discrete-element and pressure-dependent friction models	Pressure-dependent friction, sensing, and feed control; cross-machine validation remains limited	[13,14,30,33,58]
Compression/conditioning region—Conditionally applicable; no crystalline melting occurs	Empirical compression-ratio design	Energy balance and viscous dissipation	Filler-network breakdown–reorganization coupled with rheology	Reaction–thermal–mechanical coupling	[36,37,39,40,59,60]
Metering zone—Applicable; closest to the original single-screw formulation	Empirical swell and shrinkage compensation	Drag–pressure flow models	Residence-time and scorch-based quality criteria	Closed-loop online correction	[15,44,47,61,62,63,64]
Co-rotating twin-screw conveying—Conditionally applicable; element-based descriptions are preferred	Empirical equipment development	Quasi-steady slice models	Three-dimensional CFD and particle methods	Element-level monitoring and surrogate models; direct rubber-specific validation remains limited	[54,55,65,66,67]
Kneading and intermeshing region—Not applicable; no direct counterpart for elongational flow and back-mixing	Not distinguished	Not represented	Dispersive and distributive mixing indices	Prospective learned submodels; direct rubber-extrusion validation not established	[55,56]
Die and post-die relaxation—Outside the screw-zone framework	Trial-and-error die-land design	Viscoelastic swell simulation	Shape optimization	Digital-twin-oriented swell prediction; direct rubber-extrusion evidence remains limited	[15,20,61,62,68,69,70,71]

**Table 5:** Summary of the four overlapping analytical periods used to organize the development of rubber-extrusion research.

Analytical Period	Dominant Research Emphasis	Representative Advances	Evidence Limitations and Remaining Needs	References
Period I: Empirical design and equipment development (predominant before the 1980s)	Empirical operation; early analyses of conveying, pressure generation, and friction; overlapping single- and twin-screw development	Incremental advances in equipment, thermal control, and continuous-profile production	Limited material-specific constitutive modeling and validation under industrial conditions; empirical practice persisted in later periods	[13,14,15,16,30,61]
Period II: Constitutive modeling and early numerical simulation (increasing from the 1980s to the 2000s)	Generalized-Newtonian and early viscoelastic models; reduced-dimensional analysis and growing use of three-dimensional CFD	Improved calculations of pressure, temperature, residence time, and die flow	Homogeneous-material assumptions, sparse internal measurements, and geometry-specific validation	[17,18,19,20,21,68]
Period III: Engineering-scale CFD and specialized rubber models (increasing from the 2000s to 2015)	Rubber-specific rheology, viscoelastic and fractional models, multiscale concepts, and process optimization	More detailed screw and die studies, including scorch- and residence-time-aware analyses	Case-specific calibration and limited cross-machine and cross-formulation validation	[44,112,114,119,123,144]
Period IV: Multiphysics, sensing, and data-enabled methods (increasing after 2015)	Coupled simulation, online sensing, soft sensors, digital-twin concepts, PINNs, and neural operators	Selected rubber monitoring and control demonstrations, supported by broader enabling evidence from adjacent domains	Uneven rubber-specific validation; limited reporting of latency, uncertainty, and robustness under domain shift	[3,23,24,25,26,131,136,139,174]

**Table 6:** Prioritized research roadmap for rubber extrusion, with proposed deliverables and evaluation criteria.

Horizon	Objective	Concrete Deliverable	Suggested Evaluation Criterion
Near term (0–3 years)	Benchmark dataset and reporting framework	Open, versioned datasets linking formulation, equipment, operating conditions, sensor signals, rheology and product quality	Multiple machines and compound families; complete metadata, raw and processed signals, persistent identifier and reusable license
Near term (0–3 years)	Validation and uncertainty protocol	Minimum reporting standard for calibration, convergence, validation level and prediction uncertainty	Independent benchmark or interlaboratory comparison, with uncertainty, deviations and failure cases reported
Medium term (3–7 years)	Rubber-specific real-time modeling and validation	Reduced-order or learned model for coupled flow, thermal history and scorch, validated on an operating rubber-extrusion line	Validation against measured pressure, torque, temperature and product quality; end-to-end latency demonstrated
Medium term (3–7 years)	Cross-machine and cross-formulation transfer	Domain-adaptation or transfer-learning framework for machine and formulation changeover	Tested on previously unseen machines or formulations with predefined uncertainty, safety and off-spec-material criteria
Long term (beyond 7 years)	Integrated intelligent and sustainable line-level optimization	Coordinated optimization integrating quality, throughput, energy, material loss, reclaimed-rubber content and emissions	Sustained improvement over a documented production campaign, with failures, downtime and property retention reported

## Data Availability

No new data were created or analyzed in this study. Data sharing is not applicable to this article.
